# Reactivity of uranium(iii) with H_2_E (E = S, Se, Te): synthesis of a series of mononuclear and dinuclear uranium(iv) hydrochalcogenido complexes†
†Electronic supplementary information (ESI) available: Full synthetic and experimental details, spectroscopic data for ^1^H NMR, SQUID and UV/vis and detailed X-ray crystallographic data in CIF format. CCDC 1020771–1020776. For ESI and crystallographic data in CIF or other electronic format see DOI: 10.1039/c4sc02602k


**DOI:** 10.1039/c4sc02602k

**Published:** 2014-09-29

**Authors:** Sebastian M. Franke, Michael W. Rosenzweig, Frank W. Heinemann, Karsten Meyer

**Affiliations:** a Department of Chemistry and Pharmacy , Inorganic Chemistry , Friedrich-Alexander University of Erlangen-Nürnberg (FAU) , Egerlandstraße 1 , D-91058 Erlangen , Germany . Email: karsten.meyer@fau.de ; Fax: +49 9131 8527367 ; Tel: +49 9131 8527360

## Abstract

We report the syntheses, electronic properties, and molecular structures of a series of mono- and dinuclear uranium(iv) hydrochalcogenido complexes supported by the sterically demanding but very flexible, single N-anchored tris(aryloxide) ligand (^Ad^ArO)_3_N)^3–^. The mononuclear complexes [((^Ad^ArO)_3_N)U(DME)(EH)] (E = S, Se, Te) can be obtained from the reaction of the uranium(iii) starting material [((^Ad^ArO)_3_N)U^III^(DME)] in DME *via* reduction of H_2_E and the elimination of 0.5 equivalents of H_2_. The dinuclear complexes [{((^Ad^ArO)_3_N)U}_2_(μ-EH)_2_] can be obtained by dissolving their mononuclear counterparts in non-coordinating solvents such as benzene. In order to facilitate the work with the highly toxic gases, we created concentrated THF solutions that can be handled using simple glovebox techniques and can be stored at –35 °C for several weeks.

## Abstract 2

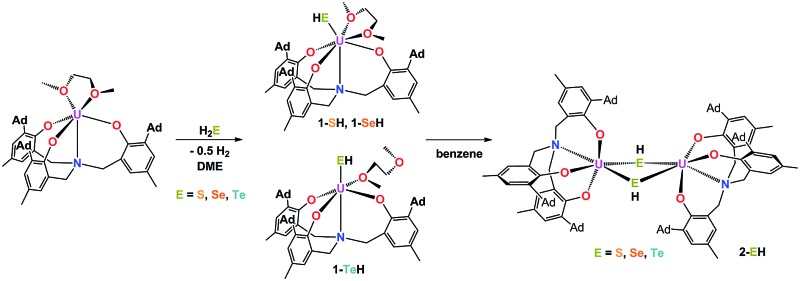
Reaction of [((^Ad^ArO)_3_N)U^III^(DME)] with EH_2_ (E = S, Se, Te) yields a complete series of mono- and dinuclear uranium(iv) hydrochalcogenide complexes.

## Introduction

Hydrochalcogenido complexes have received considerable interest in transition metal chemistry due to their potential applications as hydrodesulfurization and hydrogenation catalysts,^1–9^ as well as their relevance in bioinorganic chemistry.^1,9–15^ This class of compounds also serves as excellent precursors to a variety of homo- and hetero-bimetallic chalcogenido clusters with high nuclearity,^9,16–25^ some of which exhibit interesting optical properties.^26,27^ Furthermore, hydrochalcogenides have shown unusual reactivity such as Michael-type addition reactions with activated alkenes to afford the respective chalcogenolato complexes.^19,28,29^ In actinide chemistry, reports on chalcogenido complexes in general are rather scarce compared to the transition metals.^30–48^ Nevertheless, there has been a tremendous recent increase of interest in these types of compounds, which is motivated by the academic interest in synthesizing novel uranium–chalcogenide compounds and the fundamental importance of understanding the nature of covalent bonding between hard uranium ions and the soft chalcogenido ligands.^49–52^ In contrast to their transition metal chemistry, heavier main group 6 hydrochalcogenides of the actinides are exceedingly rare. In fact, the only reported compound is the hydrosulfido complex [(Cp*)_3_U(SH)] (Cp* = C_5_Me_5_) that was synthesized by Spirlet *et al.* from H_2_S or in a salt metathesis reaction from [(Cp*)_3_U(Br)] and NaSH, but no crystallographic evidence was provided.^53^

**Chart 1:**
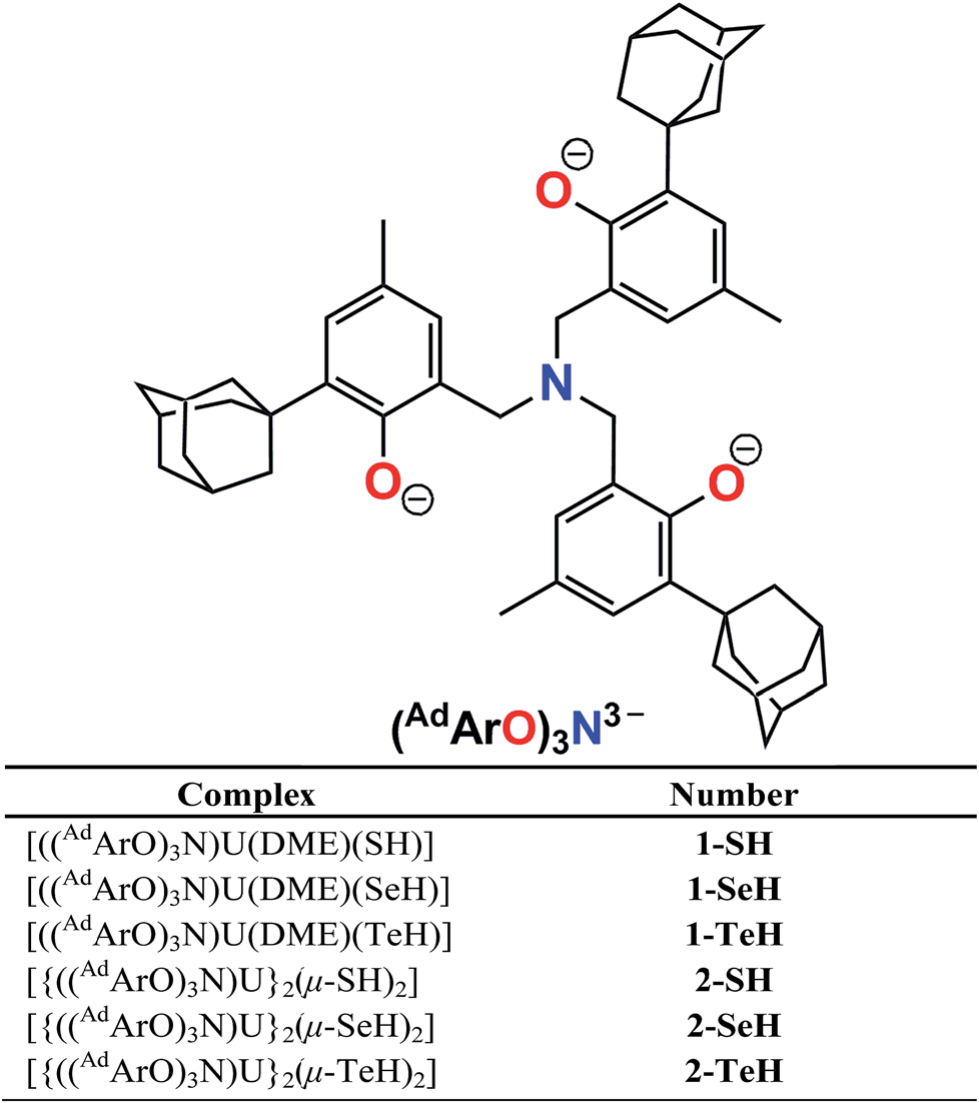
Complex formulas and numbering scheme of uranium complexes **1-EH** and **2-EH** (E = S, Se, Te), employing the chelating N-anchored ligand (^Ad^ArO)_3_N^3–^ (top).

Herein, we report the synthesis of the first series of uranium hydrochalcogenido complexes that can be obtained as mononuclear complexes [((^Ad^ArO)_3_N)U(DME)(EH)] (**1-EH**, E = S, Se, Te) or as dinuclear complexes [{((^Ad^ArO)_3_N)U}_2_(μ-EH)_2_] (**2-EH**, E = S, Se, Te) depending on the choice of solvent (Chart 1). In this manner, either **1-EH** or **2-EH** can be synthesized from the uranium(iii) starting material [((^Ad^ArO)_3_N)U(DME)] *via* reduction of H_2_E and the elimination of 0.5 equivalents of H_2_.

**Table:** 

Complex	Number
[((^Ad^ArO)_3_N)U(DME)(SH)]	**1-SH**
[((^Ad^ArO)_3_N)U(DME)(SeH)]	**1-SeH**
[((^Ad^ArO)_3_N)U(DME)(TeH)]	**1-TeH**
[{((^Ad^ArO)_3_N)U}_2_(μ-SH)_2_]	**2-SH**
[{((^Ad^ArO)_3_N)U}_2_(μ-SeH)_2_]	**2-SeH**
[{((^Ad^ArO)_3_N)U}_2_(μ-TeH)_2_]	**2-TeH**

## Results and discussion

Recently, we reported the synthesis of the uranium(iii) complex [((^Ad^ArO)_3_N)U(DME)] supported by the flexible, N-anchored chelator (^Ad^ArO)_3_N^3–^ (with (^Ad^ArO)_3_N^3–^ = tris(2-hydroxy-3-adamantyl-5-methylbenzyl)amine).^54^ This reactive, low-valent complex can undergo reactions such as the activation of CO_2_ and its heterocumulene analogs COS and CS_2_,^55–57^ as well as the activation of elemental chalcogens.^58^ The starting material [((^Ad^ArO)_3_N)U(DME)] exhibits a half-step potential of –1.879 V for the uranium(iii/iv) redox couple (see ESI†), supporting the fact that this complex is a potent reductant, which – in part – explains its observed reactivity with the H_2_E substrates. Furthermore, even uranium(iv) complexes supported by the (^Ad^ArO)_3_N^3–^ ligand can show remarkable reactivity under the right conditions, such as the formation of polychalcogenido complexes through stepwise addition of stoichiometrically precise amounts of elemental chalcogen.^59^ H_2_S and its heavier congeners H_2_Se and H_2_Te are known and well-documented precursors for the synthesis of hydrochalcogenides, however, the resultant hydrochalcogenides are rarely stable compounds and subsequent reactions often lead to the formation of metal polychalcogenide clusters or binary metal salts instead.^1,9^ Furthermore, synthetic work is greatly complicated by the high toxicity and malodor of these gases. With the commercial availability of H_2_S as a solution in THF, reactions with this gas can be carried out under much simpler conditions. That said, no such reagents exist for its selenium and tellurium analogs. To solve these synthetic challenges, we created concentrated solutions of H_2_Se and H_2_Te by condensing the respective gas formed from Al_2_E_3_ (E = Se, Te) and sulfuric acid into a THF solution at –70 °C.^60^ These solutions can be stored under an inert atmosphere in a freezer at –35 °C for several weeks. This procedure greatly simplifies handling H_2_Se and H_2_Te.

### Synthesis and molecular structures of mononuclear uranium(iv) hydrochalcogenido complexes **1-EH** (E = S, Se, Te)

**Scheme 1:**
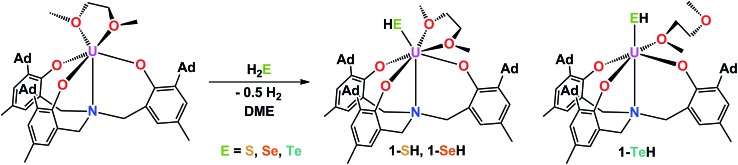
Synthesis of hydrochalcogenido complexes **1-EH** (E = S, Se, Te).

**Fig. 1:**
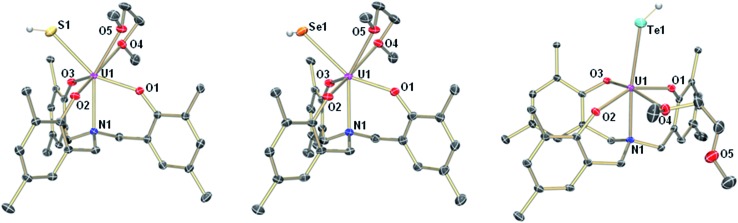
Molecular structures of uranium hydrosulfido complex **1-SH** (left), uranium hydroselenido complex **1-SeH** (center), and uranium hydrotellurido complex **1-TeH** (right). The chalcogen-bound H atoms in **1-SH** and **1-TeH** could be located in the difference Fourier map, the hydrogen in **1-SeH** was placed in a position of optimized geometry. All other H atoms, the adamantyl groups, and co-crystallized solvent molecules are omitted for clarity. Thermal ellipsoids are at 50% probability.

The mononuclear thiolato complex [((^Ad^ArO)_3_N)U(DME)(SH)] (**1-SH**) has been synthesized by reacting the uranium(iii) starting material [((^Ad^ArO)_3_N)U(DME)] in THF with an excess amount of H_2_S *via* dropwise addition of a solution of H_2_S in THF until the reaction mixture turned green. Within five minutes, a light green precipitate formed and **1-SH** was obtained in 70% yield after filtration and drying of the solids *in vacuo* (Scheme 1). Recrystallization of the solid *via* diffusion of *n*-hexane into a concentrated DME solution yielded crystals suitable for X-ray diffraction analysis. The solid state structure of **1-SH** reveals a mononuclear complex [((^Ad^ArO)_3_N)U(DME)(SH)] with a seven-coordinate uranium center in a distorted, monocapped trigonal prismatic coordination environment, in which one molecule of DME is coordinated to the uranium center in a bidentate fashion (Fig. 1, left). The U–S bond distance of 2.797(1) Å is clearly longer than reported U=S double bonds (2.382(11)–2.481(1) Å) and is in good agreement with a U–S single bond (2.588(1)–2.907(3) Å, Table 1).^58,59,61–65^ Furthermore, the chalcogen-bound hydrogen could be located in the difference Fourier map and was subsequently treated using a riding model. The U–N distance of 2.616(2) Å and the U–O_avg._ bonds of 2.171 Å are comparable to other complexes supported by the N-anchored ligand (^Ad^ArO)_3_N^3–^.^54,58,59^ Additionally, the SH^–^ ligand is not coordinated in the axial position directly *trans* to the nitrogen anchor, which is clearly shown in the N–U–S bond angle of 136.24(4)°.

**Table 1:** Selected bond distances (Å) and angles (°) for complexes **1-EH** and **2-EH** (E = S, Se, Te)

Structural parameters	**1-SH**	**1-SeH**	**1-TeH**	**2-SH**	**2-SeH**	**2-TeH**
U–O_avg._	2.169	2.170	2.130	2.121, 2.122	2.120, 2.122	2.119, 2.136
U–O_DME_	2.582(2) 2.616(2)	2.579(3) 2.599(3)	2.508(2)	—	—	—
U–N	2.616(2)	2.605(3)	2.575(2)	2.546(2), 2.542(2)	2.552(3), 2.547(3)	2.558(3), 2.567(3)
U_1_–E_1,2_	2.797(1)	2.936(1)	3.122(1)	2.882(1), 2.961(1)	2.992(1), 3.090(1)	3.145(2), 3.296(2)
U_2_–E_1,2_	—	—	—	2.964(1), 2.877(1)	3.094(1), 2.989(1)	3.163(2), 3.119(2)
N–U–E	136.24(4)	133.44(7)	173.25(4)	166.14(4), 87.79(4), 88.57(5), 166.63(4)	168.44(6), 87.25(6), 88.23(6), 169.00(6)	171.00(7), 87.43(8), 94.94(9), 166.68(10)
U–E_2_–U	—	—	—	159.99	158.10	160.09
E_1_···E_2_	—	—	—	3.888	4.157	4.479
U_1_···U_2_	—	—	—	4.295	4.381	4.448

Applying a similar synthetic procedure, the mononuclear selenolato complex [((^Ad^ArO)_3_N)U(DME)(SeH)] (**1-SeH**) can be synthesized in 72% yield (Scheme 1). The molecular structure of **1-SeH**, obtained from crystals grown from a concentrated DME solution, compares well to that of **1-SH** (Fig. 1, center). Noteworthy, the asymmetric unit of the cell in **1-SeH** contains two independent but structurally very similar molecules of the complex, and hence, only the values of **1-SeH A** are given in Table 1 (for more details see ESI†). According to the 3*σ* criterion, the U–N (2.605(3) Å) and U–O_avg._ bond lengths (2.170 Å) are the same as those observed in **1-SH**. The U–Se bond of 2.936(1) Å is significantly longer than a distinctive U=Se double bond (2.533(1)–2.646(1) Å) and can be compared to other complexes with U–Se single bond distances varying from 2.719(1) to 3.125(1) Å.^57–59,61–63,66,67^ As in complex **1-SH**, the N–U–Se angle is strongly bent with 133.44(7)°. In contrast to **1-SH**, the selenium-bound hydrogen could not be located in the difference Fourier map, but was confirmed in ^1^H and ^2^H{^1^H} NMR experiments. The ^1^H NMR spectrum of **1-SeH** recorded in pyridine-*d*_5_ shows a total of ten signals, ranging from –63 to 11 ppm, however, the unambiguous assignment of the hydrogen resonance of the SeH^–^ ligand is severely hampered due to the complicated nature of the supporting ligand system. As a result, the deuterated analog of **1-SeH**, namely [((^Ad^ArO)_3_N)U(DME)(SeD)] (**1-SeD**), was synthesized in a reaction of the uranium(iii) starting material [((^Ad^ArO)_3_N)U(DME)] with D_2_Se. As expected, the ^1^H NMR spectrum of **1-SeD** shows only nine signals, and thus, unequivocally designates the signal at –62.86 ppm to the SeH^–^ hydrogen. Additional ^2^H{^1^H} NMR experiments in pyridine revealed a single deuterium signal at –62.69 ppm that originates from the SeD^–^ moiety, further supporting the previous assignment in the ^1^H NMR spectrum and confirming the presence of the chalcogen-bound hydrogen.

In contrast to the closely related syntheses of **1-SH** and **1-SeH**, the preparation of the tellurium analog complex [((^Ad^ArO)_3_N)U(DME)(TeH)] (**1-TeH**), requires special precautions, since H_2_Te is very susceptible to light and quickly decomposes at temperatures above 0 °C. As a consequence, the reaction needs to be carried out in cooled solvents under the rigorous exclusion of light to obtain **1-TeH** in 78% yield (Scheme 1). Remarkably, the resulting complex **1-TeH** is both stable at elevated temperatures up to 80 °C and in the presence of light, which contrasts with the very few reported, usually rather unstable, transition metal complexes featuring a TeH^–^ ligand.^1,9^ Single crystals suitable for X-ray diffraction were obtained *via* diffusion of *n*-hexane into a mixture of benzene and DME (7 : 3). The molecular structure of **1-TeH** is distinctly different from those of **1-SH** and **1-SeH**, as it shows a six-coordinate uranium center, located in a distorted octahedral coordination environment (Fig. 1, right). Furthermore, and consequently, the DME solvent molecule is now coordinated in a monodentate fashion. The U–N (2.575(1) Å) and U–O_avg._ bond lengths (2.130 Å) are slightly shorter than those observed in **1-SH** and **1-SeH**. The U–Te bond distance of 3.122(1) Å is in accordance with the formulation of a U–Te single bond (Table 1).^58,61,62,67^ The TeH^–^ ligand is coordinated almost linearly *trans* to the N-anchor in the axial position and shows a N–U–Te angle of 173.25(4)°. Attempts to obtain the hydroxo complex [((^Ad^ArO)_3_N)U(DME)(OH)] from H_2_O in the same way as complexes **1-EH** lead to the formation of the bridging oxo species [{((^Ad^ArO)_3_N)U(DME)}_2_(μ-O)] instead,^54^ even in the presence of an excess amount of water. The rate of forming the hydroxo complex [((^Ad^ArO)_3_N)U(DME)(OH)] appears to be much slower than the subsequent reaction to form [{((^Ad^ArO)_3_N)U(DME)}_2_(μ-O)]. The reason why H_2_O reacts so slowly with the uranium(iii) starting material may be ascribed to the high stability of the O–H bond compared to the decreasingly stable E–H bonds in the hydrides of the heavier chalcogens.^68^

### Synthesis and molecular structures of dinuclear uranium(iv) hydrochalcogenido complexes **2-EH** (E = S, Se, Te)

**Scheme 2:**
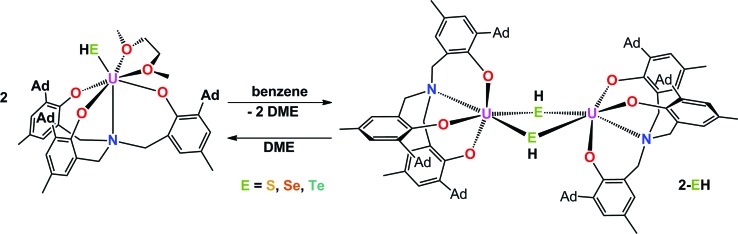
Synthesis of dinuclear hydrochalcogenido complexes **2-EH** (E = S, Se, Te).

**Fig. 2:**
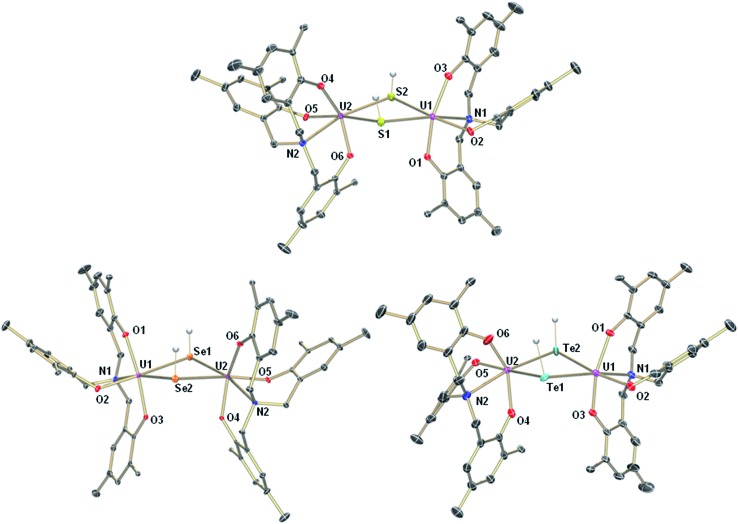
Molecular structures of uranium hydrosulfido complex **2-SH** (top), uranium hydroselenido complex **2-SeH** (bottom left), and uranium hydrotellurido complex **2-TeH** (bottom right). The chalcogen-bound H atoms in **2-SH**, **2-SeH**, and **2-TeH** could be located in the difference Fourier map. All other H atoms, the adamantyl groups and co-crystallized solvent molecules are omitted for clarity. Thermal ellipsoids are at 50% probability.

Interestingly, crystallization of **1-EH** from non-coordinating solvents, such as benzene, leads to a change in coordination geometry. X-ray diffraction analysis on crystals grown from diffusion of *n*-hexane into saturated solutions of the complexes in benzene revealed the molecular structures of the dinuclear complexes [{((^Ad^ArO)_3_N)U}_2_(μ-EH)_2_] (E = S, **2-SH**; Se, **2-SeH**; Te, **2-TeH**) (Scheme 2, Fig. 2). Each uranium center in **2-SH** is now only six-coordinate and adopts a distorted octahedral coordination geometry. The U–S bond distances of 2.878(1)–2.964(1) Å are slightly longer than in **1-SH** and are significantly longer than those observed in the dinuclear bis-μ-sulfido complex [Na(DME)_3_]_2_[{((^Ad^ArO)_3_N)U}_2_(μ-S)_2_] (2.688(2)–2.714(2) Å) or the bridging persulfido complexes [{(R_2_N)_3_U}_2_(μ-η^2^:η^2^-S_2_)] (R = SiMe_3_, 2.706(2)–2.923(2) Å) and [{((SiMe_2_NPh)_3_tacn)U}_2_(μ-η^2^:η^2^-S_2_)] (2.855(2)–2.907(3) Å).^58,63,69^ The SH^–^ ligands with the shorter U–S distances are now coordinated in the axial positions *trans* to the nitrogen anchors, which is clearly visible in the N–U–S bond angles of 166.14(4)° and 166.63(4)°. The U–O_avg._ (2.121 Å, 2.122 Å) and U–N bond distances (2.546(1) Å, 2.542(2) Å) are in good agreement with the respective structural parameters in complexes **1-EH** (E = S, Se, Te). As in complexes **1-SH** and **1-TeH**, the chalcogen-bound hydrogens in complex **2-SH** were located in the difference Fourier map.

The heavier chalcogenido complexes **2-SeH** and **2-TeH** exhibit the same molecular structures as **2-SH** and feature comparable U–O_avg._ and U–N bond lengths (see Table 1). The U–Se and U–Te bonds range from 2.989(1) to 3.094(1) Å and 3.119(2) to 3.296(2) Å, respectively, which is slightly longer compared to the bis-μ-chalcogenido complexes [Na(DME)_3_]_2_[{((^Ad^ArO)_3_N)U}_2_(μ-E)_2_] (E = Se, Te) (2.819(1)–2.866(1) Å for E = Se and 3.031(1)–3.112(1) Å for E = Te) and the bridging perselenido complex [{((^Ad^ArO)_3_N)U}_2_(μ-η^2^:η^2^-Se)(μ-DME)] (2.942(1)–3.079(1) Å).^58,59^ Furthermore, the N–U–E angles of 168.44(6)° and 169.00(6)° (**2-SeH**), as well as 171.00(7)° and 166.68(10)° (**2-TeH**) are very similar compared to **2-SH** but are slightly less bent due to a greater distance between the uranium centers (*d*(U···U) = 4.296 Å (**2-SH**) < 4.381 Å (**2-SeH**) < 4.448 Å (**2-TeH**)), which is in accordance with the larger atomic radii of selenium and tellurium that lead to slightly higher steric repulsion within the complexes. Noteworthy, the mononuclear complexes **1-EH** and their dinuclear counterparts **2-EH** showed identical ^1^H NMR spectra in deuterated benzene. Complexes supported by the highly flexible N-anchored ligand (^Ad^ArO)_3_N^3–^ as well as the often highly nucleophilic EH^–^ functional groups show a strong tendency to form dinuclear species, hence, it is within expectation that **1-EH** can dimerize to **2-EH** in non-coordinating solvents, such as benzene, with the loss of coordinating solvent.^1,54–59^ This conclusion is further supported by the presence of broadened signals at approximately 3 ppm in all spectra, which are attributed to uncoordinated DME. Likewise, the dinuclear species **2-EH** dissociates in DME to form the mononuclear complexes **1-EH**.

### Infrared spectroscopy of complexes **1-EH** and **2-EH**

IR spectroscopy can be a useful tool to determine the presence of an EH group, since the characteristic, but often rather weak IR-active *ν*_(E–H)_ bands usually appear in the regions between 2300–2600 cm^–1^ (SH),^70–76^ 2200–2500 cm^–1^ (SeH),^74–82^ and 1800–2000 cm^–1^ (TeH).^74,76,83^ However, complexes **1-SH** and **2-SH**, as well as **1-SeH**, **1-SeD** and **2-SeH**, do not show any *ν*_(E–H)_ bands at all, a fact that was also observed in several transition metal complexes.^15,18,84–90^ In contrast to this, complexes **1-TeH** and **2-TeH** both show one distinctive absorption band at 2000 cm^–1^ and 1998 cm^–1^, respectively, which is well in accordance with the scarce amount of data available for hydrotellurido complexes. Nevertheless, we could unambiguously identify the H atoms in all complexes **1-EH** and **2-EH** *via* X-ray diffraction analysis and ^2^H{^1^H} NMR spectroscopic experiments.

### UV/vis/NIR spectroscopy of complexes **1-EH** and **2-EH**

**Fig. 3:**
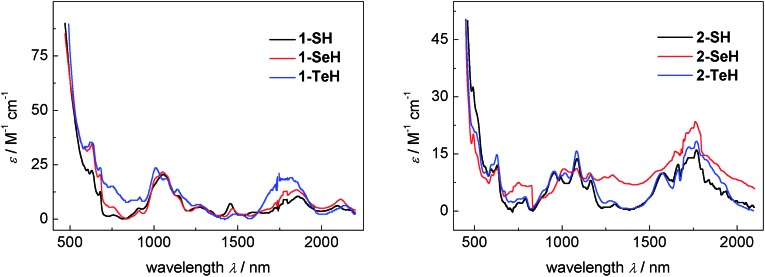
Vis/NIR spectra of hydrochalcogenido complexes **1-SH** (9.2 mM, black), **1-SeH** (9.7 mM, red), and **1-TeH** (4.1 mM, blue) recorded in DME at 25 °C (left) and vis/NIR spectra of hydrochalcogenido complexes **2-SH** (2.0 mM, black), **2-SeH** (6.6 mM, red), and **2-TeH** (6.5 mM, blue) recorded in benzene at 25 °C (right). The molar extinction coefficients for complexes **2-EH** were calculated per uranium center for a better comparison.

The vis/NIR spectra of complexes **1-EH**, recorded in DME, are all very similar and show a number of low-intensity f–f transitions with small molar extinction coefficients of 10–35 M^–1^ cm^–1^ between 500 and 2200 nm (see Fig. 3, left), characteristic for tetravalent complexes of uranium.^91^ While the spectra of **1-SH** and **1-SeH** are almost identical, the spectrum of **1-TeH** clearly shows the hypsochromic shift of two bands at 1011 nm (*ε* = 24 M^–1^ cm^–1^) and 1815 nm (*ε* = 20 M^–1^ cm^–1^). This observation can be rationalized by the discrepancy in the ligand-field splitting that should be expected due to the structural difference of **1-TeH** compared to **1-SH** and **1-SeH**. The UV/vis region shows one intense charge-transfer band centered at 287 nm for each compound with molar extinction coefficients ranging from 14–19 × 10^3^ M^–1^ cm^–1^ (see ESI, Fig. S25†). The electronic absorption spectra of complexes **2-EH**, recorded in benzene, are all very similar as well, showing very weak f–f transitions from 480 to 2000 nm (*ε* = 5–32 M^–1^ cm^–1^, Fig. 3, right) and intense charge-transfer absorptions centered at 284 nm in the UV/vis region (*ε* = 12–14 × 10^3^ M^–1^ cm^–1^, see ESI, Fig. S27†). For a better comparison, the molar extinction coefficients for complexes **2-EH** were calculated per uranium center, resulting in much lower values than those in complexes **1-EH**. This observation is indicative of weaker U–E bonds and lower covalency and is further supported by the longer U–E bonds in the molecular structures of complexes **2-EH**.^92,93^ Furthermore, in contrast to complexes **1-EH**, no hypsochromic shift was observed for any of the complexes **2-EH**, however, **2-SH** and **2-SeH** exhibit an additional charge-transfer band at 395 and 406 nm, respectively (**1-SH**, *ε* = 2185 M^–1^ cm^–1^; **1-SeH**, *ε* = 2455 M^–1^ cm^–1^), that could not be observed for **2-TeH**. The spectra of compounds **1-EH** and **2-EH** are indicative for tetravalent uranium centers and are in contrast to the spectra anticipated for a U(v) 5f^1^ or a U(iii) 5f^3^ species.^91,94–97^

### Magnetism of complexes **1-EH** and **2-EH**

**Fig. 4:**
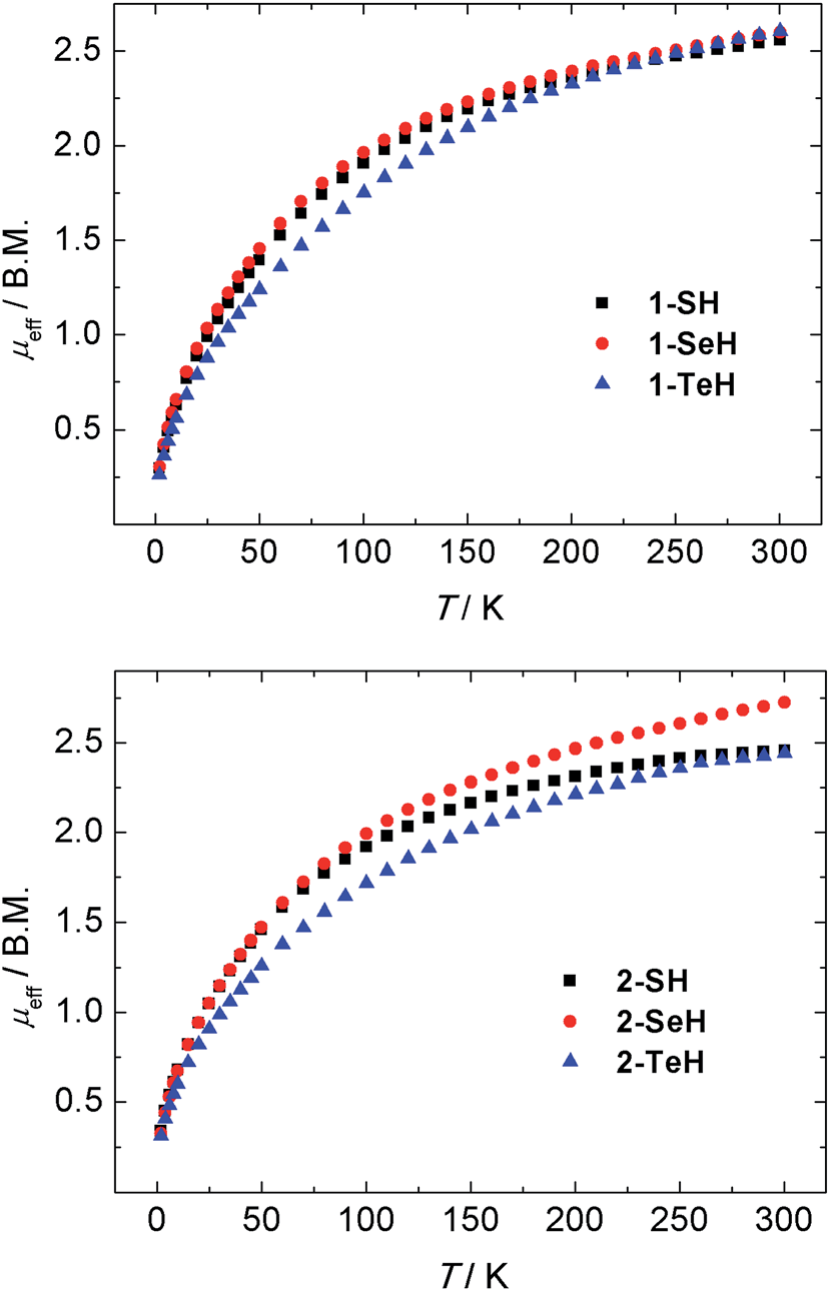
Temperature-dependent SQUID magnetization data of hydrochalcogenido complexes **1-EH** (top, E = S (black), Se (red), Te (blue)) and **2-EH** (bottom, E = S (black), Se (red), Te (blue)) as a plot of *μ*_eff_ *vs.* T. Data were corrected for underlying magnetism.

The oxidation states and electronic structures of the uranium centers in complexes **1-EH** and **2-EH** (E = S, Se, Te) were further characterized by temperature-dependent magnetic susceptibility measurements. SQUID magnetization measurements from 2–300 K of compounds **1-EH** all reveal very similar magnetic moments of 2.56 (**1-SH**), 2.60 (**1-SeH**), and 2.60 B.M. (**1-TeH**) at 300 K, which decrease with decreasing temperature to 0.30 (**1-SH**), 0.30 (**1-SeH**), and 0.26 B.M. (**1-TeH**) at 2 K (Fig. 4, top). Likewise, the dinuclear complexes **2-EH** show magnetic moments of 2.46 (**2-SH**), 2.65 (**2-SeH**), and 2.44 B.M. (**2-TeH**) at 300 K that decrease to 0.34 (**2-SH**), 0.33 (**2-SeH**), and 0.31 B.M. (**2-TeH**) at 2 K (Fig. 4, bottom). These observations are in agreement with tetravalent uranium centers with a non-magnetic ^3^H_4_ ground state.^58,98,99^ An interesting feature to note, is the difference in the plot of *χ*_M_ *vs. T* of **1-EH** and **2-EH**. While the mononuclear complexes **1-EH** show temperature-independent paramagnetism (TIP) over a wide temperature range from 10 to 62 K (for **1-SH** and **1-SeH**) and 101 K (**1-TeH**), followed by a steady decrease of the molar susceptibility, this feature is slightly less pronounced in the dinuclear complexes **2-SH** and **2-SeH** (TIP below 50 K, see ESI†) and shows a significant difference in **2-TeH** (TIP below 55 K).

## Conclusion

The reaction of H_2_E (E = S, Se, Te) with a reducing metal center is a viable synthetic route to obtain metal hydrochalcogenido complexes, however, handling of these gases is greatly complicated due to their high toxicity. With the use of concentrated and cooled solutions of these gases in THF, we were able to synthesize the new uranium(iv) hydrochalcogenido complexes [((^Ad^ArO)_3_N)U(DME)(EH)] (**1-EH**, E = S, Se, Te), using simple glovebox techniques. These compounds yield the dinuclear complexes [{((^Ad^ArO)_3_N)U}_2_(μ-EH)_2_] (**2-EH**, E = S, Se, Te) in non-coordinating solvents, such as benzene, with the loss of coordinating DME. UV/vis/NIR spectroscopy and SQUID magnetization measurements further confirm that complexes **1-EH** and **2-EH** can be clearly distinguished both in solution and in the solid state. We are currently trying to access terminal mono-chalcogenido species from the herein presented hydrochalcogenido complexes, in order to establish a series of chalcogenido complexes with U–E single and U=E double bonds with similar coordination environments. This series should provide an excellent opportunity to gain more insight into the covalency and f-orbital participation of the U–E bond in uranium chalcogenido complexes.
