# Supplementary material for: Reactivity of uranium(iii) with H2E (E = S, Se, Te): synthesis of a series of mononuclear and dinuclear uranium(iv) hydrochalcogenido complexes
Source: Chem Sci. 2014 Sep 29;6(1):275–82. doi: 10.1039/c4sc02602k (PMC5811169; doi:10.1039/c4sc02602k)
Supplement: Supplementary file 1 [file SC-006-C4SC02602K-s001.pdf]

Electronic Supplementary Material (ESI) for Chemical Science.
This journal is © The Royal Society of Chemistry 2014

Supporting Information for

Reactivity of uranium(III) with H_2E (E = S, Se, Te): Synthesis of a series of mononuclear and dinuclear uranium(IV) hydrochalcogenido complexes

*Sebastian M. Franke, Michael W. Rosenzweig, Frank W. Heinemann, and Karsten Meyer**

Department of Chemistry and Pharmacy, Inorganic Chemistry, Friedrich-Alexander University of Erlangen-Nürnberg (FAU), Egerlandstrasse 1, 91058 Erlangen, Bavaria, Germany

Table of contents

Experimental Section S3

General Methods S3

Synthesis of $[((^{Ad}ArO)_3N)U(DME)(SH)]$ (**1-SH**) S4

Synthesis of $[((^{Ad}ArO)_3N)U(DME)(SeH)]$ (**1-SeH**) S4

Synthesis of $[((^{Ad}ArO)_3N)U(DME)(SeD)]$ (**1-SeD**) S4

Synthesis of $[((^{Ad}ArO)_3N)U(DME)(TeH)]$ (**1-TeH**) S4

Synthesis of $[\{((^{Ad}ArO)_3N)U\}_2(\mu\text{-SH})_2]$ (**2-SH**) S5

Synthesis of $[\{((^{Ad}ArO)_3N)U\}_2(\mu\text{-SeH})_2]$ (**2-SeH**) S5

Synthesis of $[\{((^{Ad}ArO)_3N)U\}_2(\mu\text{-TeH})_2]$ (**2-TeH**) S5

X-Ray Crystal Structure Determination S6

Crystallographic Details S6

Crystal Data and Structure Refinement for **1-SH** S9

Crystal Data and Structure Refinement for **1-SeH** S10

Crystal Data and Structure Refinement for **1-TeH** S12

Crystal Data and Structure Refinement for **2-SH** S14

Crystal Data and Structure Refinement for **2-SeH** S15

Crystal Data and Structure Refinement for **2-TeH** S18

^{1}H NMR Spectroscopy S19

^{2}D NMR Spectroscopy S22

^{77}Se NMR Spectroscopy S23

^{125}Te NMR Spectroscopy S23

SQUID Magnetization Data S24

IR Spectroscopy S26

UV/vis Spectroscopy S29

Cyclic Voltammetry S31

References S32

Experimental Section

General Methods. All experiments were performed under a dry nitrogen atmosphere using standard Schlenk techniques or an MBraun inert-gas glovebox. All solvents were purified using a two-column solid-state purification system (Glasscontour System, Irvine, CA) and transferred to the glovebox without exposure to air, unless otherwise indicated.

Magnetism data of crystalline powdered samples (20 – 30 mg) were recorded with a SQUID magnetometer (Quantum Design) at 10 kOe (2 – 300 K for **1-EH** and **2-EH**; E = S, Se, Te). Values of the magnetic susceptibility were calculated per uranium center and corrected for the underlying diamagnetic increment (χ_{dia} = -663.7×10^{-6} cm^3mol^{-1} (**1-SH**), -671.7×10^{-6} cm^3mol^{-1} (**1-SeH**), -696.0×10^{-6} cm^3mol^{-1} (**1-TeH**), -601.2×10^{-6} cm^3mol^{-1} (**2-SH**), -609.2×10^{-6} cm^3mol^{-1} (**2-SeH**), -623.5×10^{-6} cm^3mol^{-1} (**2-TeH**)) by using tabulated Pascal constants and the effect of the blank sample holders (gelatin capsule/ straw).[1] Samples used for magnetization measurements were recrystallized multiple times and checked for chemical composition and purity by elemental analysis (C, H, N and S) and ^1H NMR spectroscopy. Data reproducibility was also carefully checked on independently synthesized samples.

^1H NMR spectra were recorded on JEOL 270 and 400 MHz instruments, operating at the respective frequencies of 269.714 and 400.178 MHz with a probe temperature of 23 °C in benzene-d_6. Chemical shifts were referenced to *protio* solvent impurities (δ = 7.16 (benzene-d_6), δ = 8.74 (pyridine-d_5)) and are reported in parts per million (ppm). Furthermore, additional NMR spectra were recorded on a Varian VNMRS 400 (^1H 399.5 MHz; $^2H\{^1H\}$ 61.32 MHz; $^{77}Se\{^1H\}$ 66.21 MHz; $^{125}Te\{^1H\}$ 126.0 MHz) spectrometer. The spectra were referenced internally to the residual protic solvent (^1H) or the naturally occurring deuterium in the protic solvent (^2H). $^{77}Se\{^1H\}$ NMR spectra were referenced externally relative to Me_2Se. $^{125}Te\{^1H\}$ NMR spectra were referenced externally relative to Me_2Te. All spectra were recorded at 303 K unless otherwise stated. Results from elemental analysis were obtained from the Analytical Laboratories at the Friedrich-Alexander-University Erlangen-Nürnberg (Erlangen, Germany) on Euro EA 3000.

Starting Materials. Precursor complexes [$(UI_3(dioxane)_{1.5}$] and [$U(N(SiMe_3)_2)_3$] were prepared as described by Kiplinger and coworkers.[2] The tris(2-hydroxy-3-adamantyl-5-methylbenzyl)amine ligand was synthesized from a modified literature procedure.[3] The uranium starting material [$((^{Ad}ArO)_3N)U(DME)$] was synthesized according to literature procedures.[4] Aluminium selenide (≥ 99 %) was purchased from Alfa Aesar, aluminum telluride (≥ 99.5 %) was purchased from Materion and both were used as received. Anhydrous 1,2-dimethoxyethane (≥ 99 %), THF (≥ 99.9 %), and benzene (99.8 %) were purchased from Sigma-Aldrich. THF and 1,2-dimethoxyethane were further dried by distilling over sodium benzophenone. Hydrogen sulfide (0.8 M in THF) was purchased from Sigma-Aldrich and used without further purification. Hydrogen selenide, deuterium selenide, and

hydrogen telluride were synthesized from Al_2E_3 (E = Se, Te) and dried according to a modified literature procedure[5] and were condensed into THF in a schlenk flask at –70 °C. In order to avoid hydrogen chloride contamination of the THF solution, a 4 M solution of H_2SO_4 in degassed water was used for the preparation of H_2Se and H_2Te instead. Additionally, the gas was passed through a cooling trap that was kept at –10 °C from the outside, instead of drying with $CaCl_2$ and P_4O_{10}. All three gases were stored under an inert atmosphere at –35 °C. It should be noted that – in contrast to H_2Se and D_2Se – H_2Te cannot be stored indefinitely under these conditions and decomposes within two to three weeks. **Warning! H_2S, H_2Se, and H_2Te are extremely toxic and odoriferous. Any reactions involving these gases should be carried out in a well-ventilated fumehood or glovebox.**

Synthesis of [((^{Ad}ArO)$_3$N)U(DME)(SH)] (1-SH). A vial was charged with [((^{Ad}ArO)$_3$N)U(DME)] (100 mg, 0.09 mmol) in THF and a solution of H_2S in THF (0.8 M, 0.09 mmol, 112 μmol) was added dropwise. The solution immediately turned green and after 2 min. a green precipitate formed. The reaction mixture was stirred for another 1 h, then filtered and the volatiles were removed *in vacuo*. Recrystallization from diffusing *n*-hexane into a concentrated DME solution yielded the pure product. Yield: 72 mg (0.063 mmol, 70%). ^{1}H NMR (270 MHz, C_5D_5N): δ [ppm] = 9.78, 8.32, 3.53, 3.31, 3.17, 2.65, 2.32, –15.21, –41.80 (1H, S*H*). Elemental analysis (%) calcd. for [((^{Ad}ArO)$_3$N)U(DME)(SH)] , C 61.20, H 6.82, N 1.23, S 2.82; found C 61.47, H 7.21, N 1.24, S 2.39.

Synthesis of [((^{Ad}ArO)$_3$N)U(DME)(SeH)] (1-SeH). A vial was charged with [((^{Ad}ArO)$_3$N)U(DME)] (100 mg, 0.09 mmol) in THF and a solution of H_2Se in THF was added dropwise until the solution turned green. After 2 min. a green precipitate formed and the reaction mixture was stirred for another 1 h, then filtered and the volatiles were removed *in vacuo*. Recrystallization from diffusing *n*-hexane into a concentrated DME solution yielded the pure product. Yield: 76.5 mg (0.070 mmol, 78%). ^{1}H NMR (270 MHz, C_5D_5N): δ [ppm] = 10.13, 9.58, 3.51, 3.46, 3.30, 2.87, 2.43, 2.33, –18.36, –62.86 (1H, Se*H*). ^{77}Se{^{1}H} NMR (67 MHz, C_5D_5N): δ [ppm] = 813.9 (1 Se, U–*Se*). Elemental analysis (%) calcd. for [((^{Ad}ArO)$_3$N)U(DME)(SeH)] , C 58.78, H 6.55, N 1.18; found C 58.89, H 6.94, N 1.11.

Synthesis of [((^{Ad}ArO)$_3$N)U(DME)(SeD)] (1-SeD). A vial was charged with [((^{Ad}ArO)$_3$N)U(DME)] (100 mg, 0.09 mmol) in THF and a solution of D_2Se in THF was added dropwise until the solution turned green. After 2 min. a green precipitate formed and the reaction mixture was stirred for another 1 h, then filtered and the volatiles were removed *in vacuo*. Yield: 56 mg (0.047 mmol, 52%). ^{1}H NMR (270 MHz, C_5D_5N): δ [ppm] = 10.17, 9.72, 3.52, 3.49, 3.29, 2.91, 2.44, 2.33, –19.12. Elemental analysis (%) calcd. for [((^{Ad}ArO)$_3$N)U(DME)(SeD)] × THF , C 59.18, H 6.89, N 1.11; found C 59.42, H 6.66, N 1.33.

Synthesis of [((^{Ad}ArO)$_3$N)U(DME)(TeH)] (1-TeH). A vial was charged with [((^{Ad}ArO)$_3$N)U(DME)] (100 mg, 0.09 mmol) in THF and cooled to –35 °C. A solution of H_2Te in cold THF was then added until the solution turned green. The reaction mixture was stirred for another 2 min. and the volatiles were removed *in vacuo*. The crude product was dissolved in DME and stirred for 15 minutes, during which a light green precipitate formed. The reaction mixture was filtered and dried *in* vacuo. Recrystallization from diffusing *n*-hexane into a concentrated benzene/DME (70:30) solution yielded the pure product. Yield: 48.6 mg (0.040 mmol, 44 %). ^{1}H NMR (270 MHz, C_5D_5N): δ [ppm] = 20.26, 11.53, 10.70, 4.01, 3.51, 3.36, 3.28, 2.75, 2.48, 2.38, 1.23, –23.28, –85.49 (1H, Te*H*). ^{125}Te{^{1}H} NMR (126 MHz, C_5D_5N): δ [ppm] = 552.5 (1 Te, U–*Te*). Elemental analysis (%) calcd. for [((^{Ad}ArO)$_3$N)U(DME)(TeH)] , C 56.46, H 6.29, N 1.14; found C 56.42, H 6.29, N 1.21.

Synthesis of [{((^{Ad}ArO)$_3$N)U}$_2$(μ-SH)$_2$] (2-SH). A vial was charged with [((^{Ad}ArO)$_3$N)U(DME)] (100 mg, 0.09 mmol) in THF and a solution of H_2S in THF (0.8 M, 0.09 mmol, 112 μmol) was added dropwise. The solution immediately turned green and after 2 min. a green precipitate formed. The reaction mixture was stirred for another 1 h, then filtered and the volatiles were removed *in vacuo*. Recrystallization from diffusing *n*-hexane into a concentrated benzene solution yielded the pure product. Yield: 67.5 mg (0.064 mmol, 72 %). ^{1}H NMR (270 MHz, C_6D_6): δ [ppm] = 60.43, 56.41, 33.21, 17.25, 16.46, 11.68, 10.36, –0.44, –1.01, –10.20, –12.05, –15.61, –17.71, –31.99, –50.05, –52.75, –79.70. Elemental analysis (%) calcd. for [{((^{Ad}ArO)$_3$N)U}$_2$(μ-SH)$_2$] × C_6H_6, C 62.97, H 6.49, N 1.26, S 2.95; found C 62.92, H 6.40, N 1.26, S 2.14.

Synthesis of [{((^{Ad}ArO)$_3$N)U}$_2$(μ-SeH)$_2$] (2-SeH). A vial was charged with [((^{Ad}ArO)$_3$N)U(DME)] (100 mg, 0.09 mmol) in THF and a solution of H_2Se in THF was added dropwise until the solution turned green. After 2 min. a green precipitate formed and the reaction mixture was stirred for another 1 h, then filtered and the volatiles were removed *in vacuo*. Recrystallization from diffusing *n*-hexane into a concentrated benzene solution yielded the pure product. Yield: 76.4 mg (0.065 mmol, 72 %). ^{1}H NMR (270 MHz, C_6D_6): δ [ppm] = 64.29, 60.12, 35.11, 18.40, 17.62, 12.47, 11.12, –0.82, –1.86, –11.28, –12.90, –16.64, –18.98, –34.09, –53.26, –55.15, –82.51. Elemental analysis (%) calcd. for [((^{Ad}ArO)$_3$N)U(DME)(SeH)] , C 59.23, H 6.17, N 1.28; found C 59.57, H 5.97, N 1.29.

Synthesis of [{((^{Ad}ArO)$_3$N)U}$_2$(μ-TeH)$_2$] (2-TeH). A vial was charged with [((^{Ad}ArO)$_3$N)U(DME)] (100 mg, 0.09 mmol) in THF and cooled to –35 °C. A solution of H_2Te in cold THF was then added until the solution turned green. The reaction mixture was stirred for another 2 min. and the volatiles were removed *in vacuo*. The crude product was dissolved in DME and stirred for 15 minutes, during which a light green precipitate formed. The reaction mixture was filtered and dried *in vacuo*. Recrystallization from diffusing *n*-hexane into a concentrated benzene solution yielded the pure product. Yield: 59 mg (0.048 mmol, 53 %). ^{1}H NMR (270 MHz, C_6D_6): δ [ppm] = 69.02, 64.95, 37.53,

19.53, 18.84, 13.30, 12.10, 5.36, 2.96, –0.52, –1.65, –3.17, –12.96, –13.89, –17.63, –20.45, –36.13, –57.18, –58.20, –85.58. Elemental analysis (%) calcd. for [{((^{Ad}ArO)$_3$N)U}$_2$(μ-TeH)$_2$] × C_6H_6, C 57.88, H 5.97, N 1.18; found C 57.94, H 6.26, N 1.08.

X-Ray Crystal Structure Determinations

CCDC-1020771 (for **1-SH**), CCDC-1020772 (for **1-SeH**), CCDC-1020773 (for **1-TeH**), CCDC-1020774 (for **2-SH**), CCDC-1020775 (for **2-SeH**), and CCDC-1020776 (for **2-TeH**) contain the supplementary crystallographic data for this paper. This data can be obtained free of charge via http://www.ccdc.cam.ac.uk/products/csd/request/
(or from Cambridge Crystallographic Data Centre, 12 Union Road, Cambridge, CB2 1EZ, UK. fax: ++44-1223-336-033; e-mail: deposit@ccdc.cam.ac.uk).

Crystallographic Details. Green prisms of complexes **1-SH** and **1-SeH** grown from diffusion of *n*-hexane into a concentrated solution of DME, green blocks of **1-TeH** grown from diffusion of n-hexane into a concentrated benzene/DME (70:30) solution, and green to yellow blocks of complexes **2-EH** (E = S, Se, Te) grown from diffusion of *n*-hexane into a concentrated benzene solution were coated with isobutylene oil on a microscope slide. Intensity data were collected using MoK_α radiation (λ = 0.71073 Å) either on a Bruker-Nonius KappaCCD (graphite monochromator) for compounds **1-SH** (150 K), **2-SeH** (100 K), and **2-TeH** (150 K), or at 100 K on a Bruker-SMART APEX2 (graphite monochromator) for compounds **1-SeH**, **1-TeH**, and **2-SH**. Data were corrected for Lorentz and polarization effects, semi-empirical absorption corrections were performed on the basis of multiple scans using *SADABS*.[6] All structures were solved by direct methods and refined by full-matrix least-squares procedures on F^2 using *SHELXTL NT* 6.12.[7] If not stated otherwise, all hydrogen atoms were placed in positions of optimized geometry, their isotropic displacement parameters were tied to those of the corresponding carrier atoms by a factor of 1.2 or 1.5.

Compound **1-SH** crystallized with one molecule of DME per formula unit. The position of the sulphur bound H atom H1S was derived from a difference fourier synthesis and allowed to ride on the S atom. All other hydrogen atoms were placed in positions of optimized geometry.

The asymmetric unit of the cell in compound **1-SeH** contained two independent molecules of the complex and two molecules of benzene. Both solvent molecules were disordered. Two alternative orientations were refined resulting in site occupancies of 32.9(6) and 67.1(6) % for the atoms C201 – C206 and C211 – C216, respectively and of 56.5(6) and 43.5(6) % for the atoms C301 – C306 and C311 – C316. SIMU restraints were applied in the refinement of the disordered benzenes.

The tentative position of the Te bound hydrogen H1 in compound **1-TeH** was taken from a difference fourier and it was subsequently treated using a riding model. All other hydrogen atoms were placed in positions of optimized geometry.

Compound **2-SH** crystallized with a total of 2.25 molecules of benzene and half a molecule of *n*-hexane. The 0.25 benzene is situated on a crystallographic inversion center and is occupied by only 50 %. The *n*-hexane molecule is situated on a crystallographic inversion center. The tentative position of the S bound hydrogen atoms H1S and H2S were taken from a difference fourier and they were subsequently treated using a riding model. All other hydrogen atoms were placed in positions of optimized geometry.

Compound **2-SeH** crystallized with a total of 2.5 molecules of benzene and 0.5 molecules of *n*-hexane. The two half molecules were situated on crystallographic inversion centres. SIMU and ISOR restraints were applied in the refinement of the half benzene molecule (C401 – C403). Treatment of hydrogen atoms: The positions of the two hydrogen atoms attached to the Se atoms were derived from a difference fourier synthesis and their positional parameters were refined. All other hydrogen atoms were placed in positions of optimized geometry.

The dinuclear complex molecule **2-TeH** was strongly disordered around one uranium centre. Two alternative orientations of the ligand around U2 were refined, including two alternative sites for U2, Te1 and Te2. The corresponding site occupancies turned out to be 50 % each. The compound crystallized with a total of 6.65 molecules of benzene, 0.35 molecules of *n*-hexane, and 0.35 of a putative water molecule. One half benzene molecule was situated on a crystallographic inversion centre (C401 – C403), another benzene was occupied by only 50 % (C901 – C906). One benzene molecule (C801 – C806) shares its site with a *n*-hexane molecule (C811 – C816) in a ratio of 65 : 35 %. One remaining significant residual electron density maximum was attributed to a putative water molecule of 35 % occupancy as the compound was extremely sensitive to moisture and air. DFIX restraints were applied in the refinement of the Te – H and the O – H distances. SIMU and ISOR restraints were applied in the refinement of the disordered parts of the complex molecule as well as for the solvent molecules. Treatment of hydrogen atoms: The positions of the two hydrogen atoms attached to the Te atoms were derived from a difference fourier synthesis as well as tentative positions for the hydrogen atoms of the putative water molecule. These hydrogens were subsequently refined using a riding model.

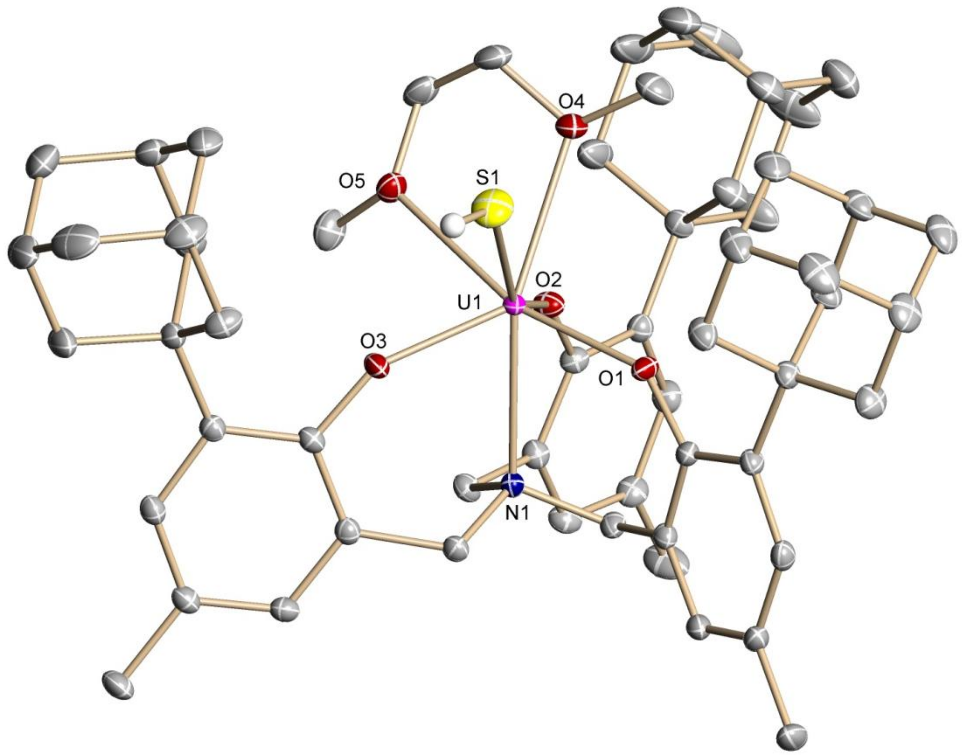

Figure S1. Molecular structure of **1-SH** with atom numbering scheme. Non-chalcogen bound H-atoms and co-crystallized solvent molecules are omitted for clarity. Thermal ellipsoids are at 50% probability.

Crystal data and structure refinement for 1-SH.

Identification code	sf01
Empirical formula	C62 H87 N O7 S U
Formula weight	1228.42 g/mol
Temperature	150(2) K
Wavelength	0.71073 Å
Crystal system, space group	Triclinic, P-1
Unit cell dimensions	a = 14.4228(2) Å α = 112.831(3)°<br>b = 14.8678(5) Å β = 96.488(4)°<br>c = 15.4212(9) Å γ = 108.933(2)°
Volume	2772.59(19) $Å^3$
Z, Calculated density	2, 1.471 Mg/m^3
Absorption coefficient	3.019 mm^{-1}
F(000)	1260
Crystal size	0.30 x 0.28 x 0.11 mm^3
Theta range for data collection	3.80 to 28.70°
Limiting indices	–19<=h<=19, –20<=k<=20, –20<=l<=20
Reflections collected / unique	112535 / 14282 [R(int) = 0.0318]
Completeness to theta = 28.70	99.7 %
Absorption correction	Semi-empirical from equivalents
Max. and min. transmission	0.746 and 0.554
Refinement method	Full-matrix least-squares on F^2
Data / restraints / parameters	14282 / 0 / 656
Goodness-of-fit on F^2	1.153
Final R indices [I>2sigma(I)]	R1 = 0.0189, wR2 = 0.0450
R indices (all data)	R1 = 0.0261, wR2 = 0.0492
Largest diff. peak and hole	2.098 and –1.042 $e.A^{-3}$

Crystal data and structure refinement for 1-SeH.

Identification code	sf02
Empirical formula	C64 H83 N O5 Se U
Formula weight	1263.30 g/mol
Temperature	100(2) K
Wavelength	0.71073 Å
Crystal system, space group	Triclinic, P-1
Unit cell dimensions	a = 16.827(1) Å α = 88.022(3)°<br>b = 17.007(1) Å β = 80.746(3)°<br>c = 19.804(2) Å γ = 82.903(3)°
Volume	5550.0(6) $Å^3$
Z, Calculated density	4, 1.512 Mg/m^3
Absorption coefficient	3.631 mm^{-1}
F(000)	2560
Crystal size	0.52 x 0.30 x 0.22 mm^3
Theta range for data collection	2.58 to 27.10°
Limiting indices	–21<=h<=21, –21<=k<=21, –25<=l<=25
Reflections collected / unique	158317 / 24462 [R(int) = 0.0439]
Completeness to theta = 27.10	99.8 %
Absorption correction	Semi-empirical from equivalents
Max. and min. transmission	0.421 and 0.305
Refinement method	Full-matrix least-squares on F^2
Data / restraints / parameters	24462 / 252 / 1371
Goodness-of-fit on F^2	1.047
Final R indices [I>2sigma(I)]	R1 = 0.0354, wR2 = 0.0813
R indices (all data)	R1 = 0.0463, wR2 = 0.0867
Largest diff. peak and hole	4.391 and –1.842 e. $Å^{-3}$

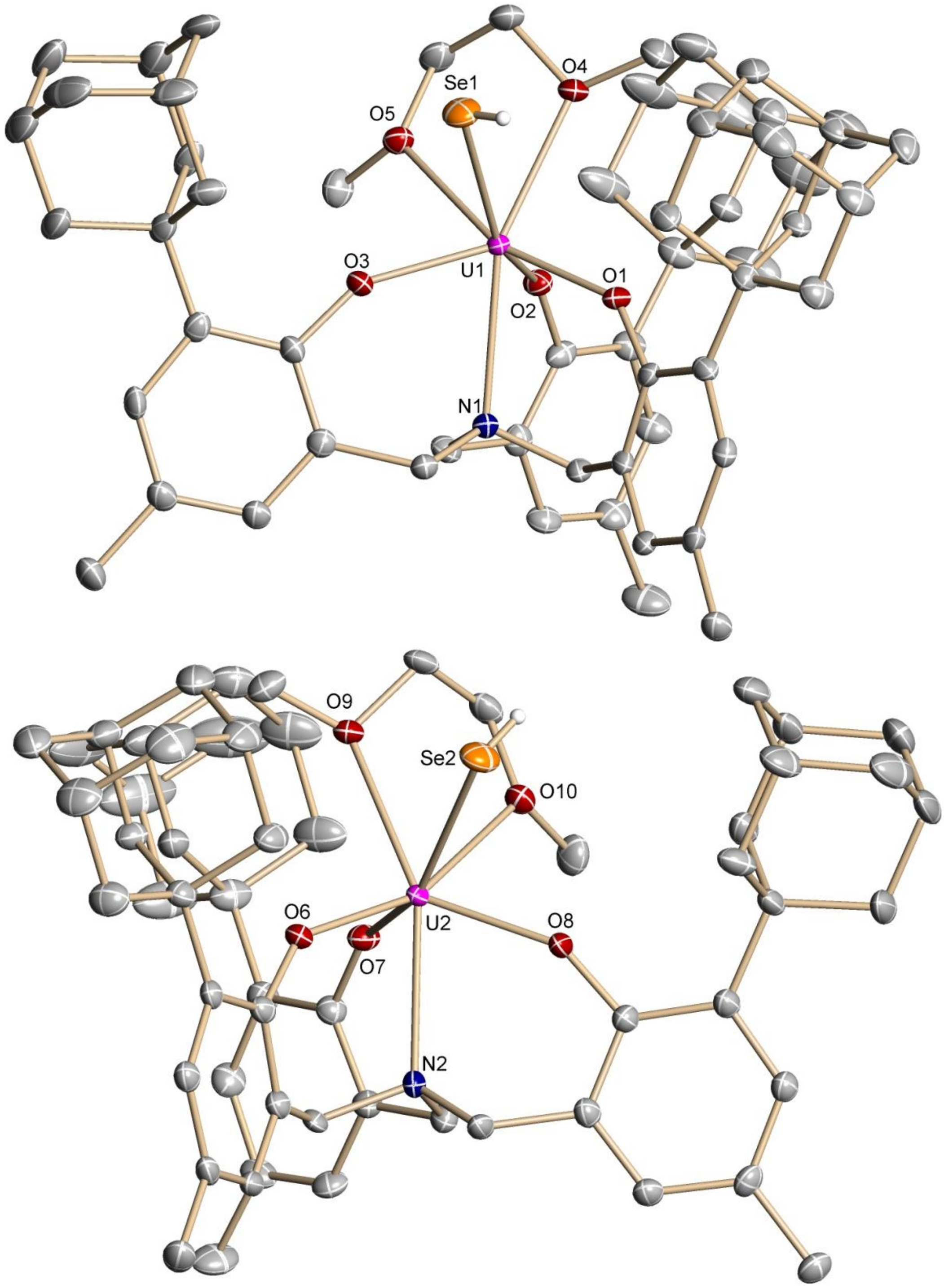

Figure S2. Molecular structures of two crystallographically independent molecules of **1-SeH** (**A**, top; **B**, bottom) with atom numbering scheme. Non-chalcogen bound H-atoms and co-crystallized solvent molecules are omitted for clarity. Thermal ellipsoids are at 50% probability.

Table S1. Selected bond distances (Å) and angles (°) for complexes **1-SeH A** and **1-SeH B**.

Structural Parameters	1-SeH A	1-SeH B
U–$O_{avg.}$	2.170	2.161
U–O_{DME}	2.599(3), 2.579(3)	2.598(3), 2.577(3)
U–N	2.605(3)	2.606(3)
U–E	2.936(1)	2.936(1)
N–U–E	133.44(7)	132.57(7)

Crystal data and structure refinement for 1-TeH.

Identification code	sf03
Empirical formula	C58 H77 N O5 Te U
Formula weight	1233.84 g/mol
Temperature	100(2) K
Wavelength	0.71073 Å
Crystal system, space group	Triclinic, P-1
Unit cell dimensions	a = 11.2439(15) Å α = 102.273(4)°<br>b = 12.9994(17) Å β = 101.519(4)°<br>c = 18.636(3) Å γ = 100.806(4)°
Volume	2531.8(6) $Å^3$
Z, Calculated density	2, 1.618 Mg/m^3
Absorption coefficient	3.820 mm^{-1}
F(000)	1232
Crystal size	0.45 x 0.20 x 0.16 mm^3
Theta range for data collection	2.20 to 28.70°
Limiting indices	–15<=h<=15, –17<=k<=17, –25<=l<=25
Reflections collected / unique	152787 / 13024 [R(int) = 0.0310]
Completeness to theta = 27.10	99.9 %
Absorption correction	Semi-empirical from equivalents
Max. and min. transmission	0.746 and 0.522
Refinement method	Full-matrix least-squares on F^2
Data / restraints / parameters	13024 / 0 / 600
Goodness-of-fit on F^2	1.076
Final R indices [I>2sigma(I)]	R1 = 0.0205, wR2 = 0.0514
R indices (all data)	R1 = 0.0215, wR2 = 0.0518
Largest diff. peak and hole	2.976 and –1.403 e. $Å^{-3}$

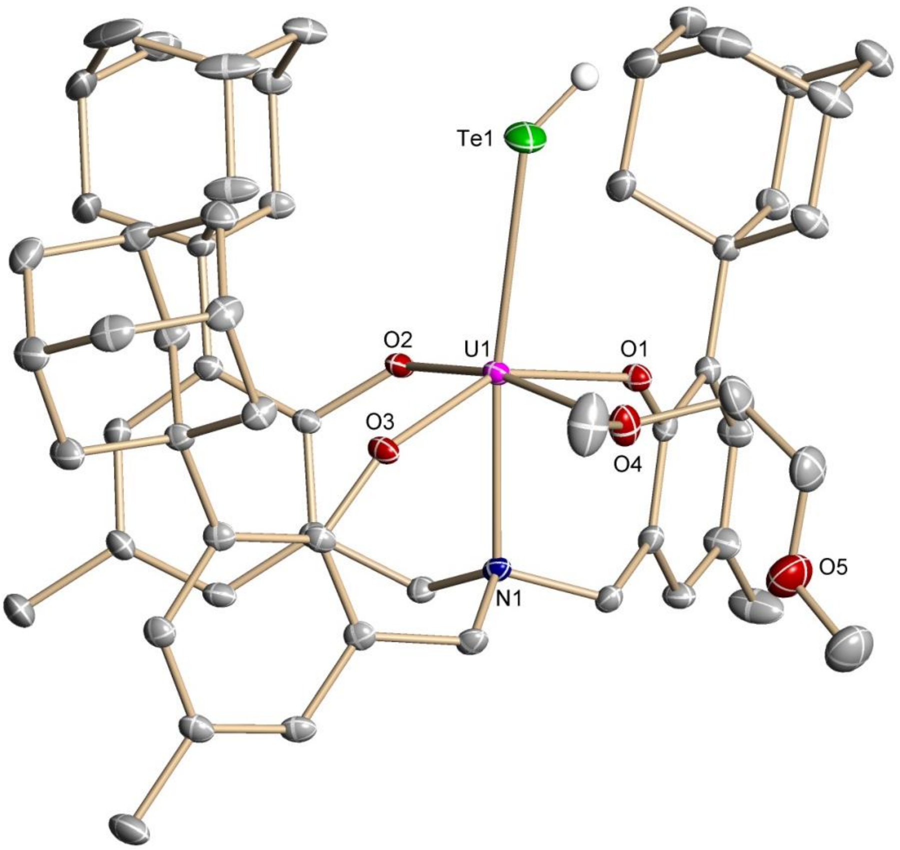

Figure S3. Molecular structures of **1-TeH** (top) and **2-SH** (bottom) with atom numbering scheme. Non-chalcogen bound H-atoms and co-crystallized solvent molecules are omitted for clarity. Thermal ellipsoids are at 50% probability.

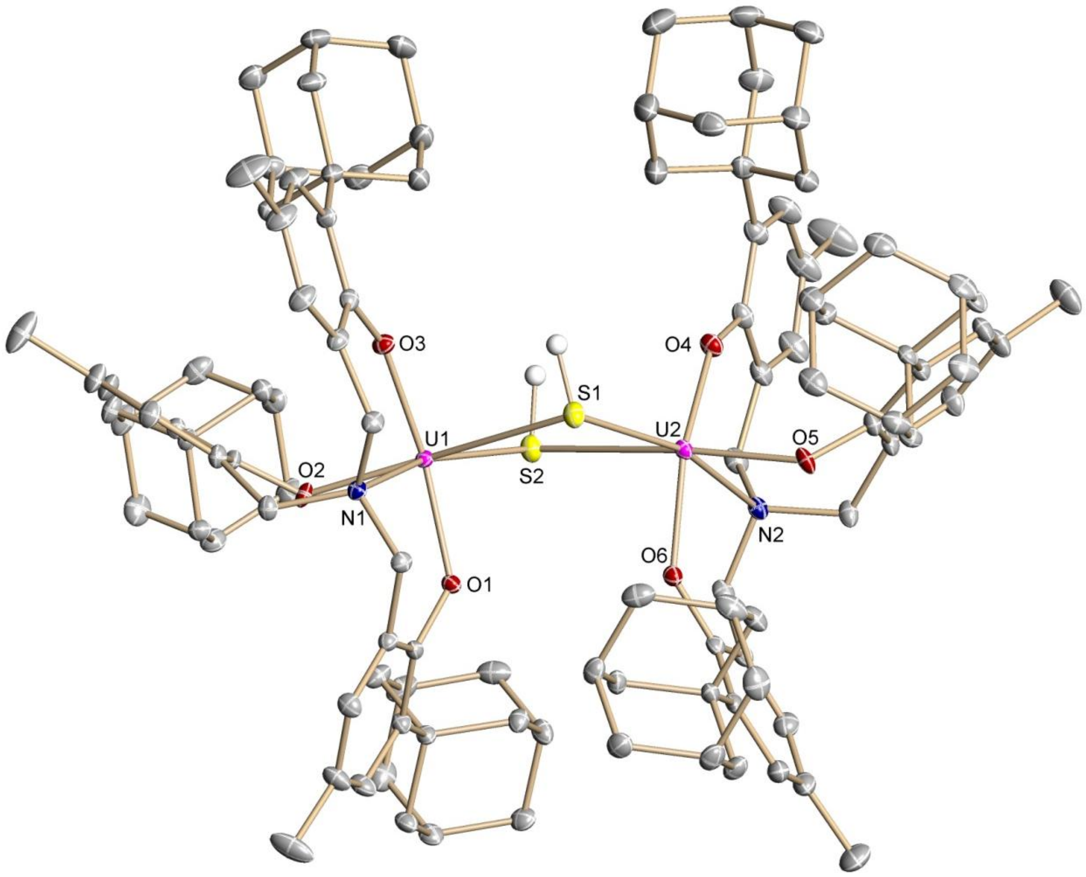

Crystal data and structure refinement for 2-SH.

Identification code	sf04
Empirical formula	C124.50 H154.50 N2 O6 S2 U2
Formula weight	2315.18 g/mol
Temperature	100(2) K
Wavelength	0.71073 Å
Crystal system, space group	Triclinic, P-1
Unit cell dimensions	a = 11.1756(16) Å α = 85.233(5)°<br>b = 16.817(2) Å β = 87.451(5)°<br>c = 28.662(4) Å γ = 88.366(5)°
Volume	5361.0(13) $Å^3$
Z, Calculated density	2, 1.434 Mg/m^3
Absorption coefficient	3.112 mm^{-1}
F(000)	2359
Crystal size	0.40 x 0.26 x 0.14 mm^3
Theta range for data collection	1.22 to 29.80°
Limiting indices	–15<=h<=15, –23<=k<=23, –39<=l<=39
Reflections collected / unique	334803 / 30030 [R(int) = 0.0427]
Completeness to theta = 27.00	99.9 %
Absorption correction	Semi-empirical from equivalents
Max. and min. transmission	0.746 and 0.587
Refinement method	Full-matrix least-squares on F^2
Data / restraints / parameters	30030 / 3 / 1232
Goodness-of-fit on F^2	1.100
Final R indices [I>2sigma(I)]	R1 = 0.0257, wR2 = 0.0608
R indices (all data)	R1 = 0.0321, wR2 = 0.0629
Largest diff. peak and hole	1.937 and –1.875 e. $Å^{-3}$

Crystal data and structure refinement for 2-SeH.

Identification code	sf05
Empirical formula	C126 H156 N2 O6 Se2 U2
Formula weight	2428.51 g/mol
Temperature	100(2) K
Wavelength	0.71073 Å
Crystal system, space group	Triclinic, P-1
Unit cell dimensions	a = 11.217(2) Å α = 85.324(9)°<br>b = 16.815(3) Å β = 87.434(9)°<br>c = 28.680(3) Å γ = 88.412(16)°
Volume	5384.5(15) $Å^3$
Z, Calculated density	2, 1.498 Mg/m^3
Absorption coefficient	3.737 mm^{-1}
F(000)	2452
Crystal size	0.26 x 0.20 x 0.12 mm^3
Theta range for data collection	2.87 to 28.50°
Limiting indices	–15<=h<=15, –22<=k<=22, –38<=l<=38
Reflections collected / unique	105025 / 27195 [R(int) = 0.0418]
Completeness to theta = 27.00	99.8 %
Absorption correction	Semi-empirical from equivalents
Max. and min. transmission	0.746 and 0.626
Refinement method	Full-matrix least-squares on F^2
Data / restraints / parameters	27195 / 30 / 1257
Goodness-of-fit on F^2	1.075
Final R indices [I>2sigma(I)]	R1 = 0.0335, wR2 = 0.0656
R indices (all data)	R1 = 0.0489, wR2 = 0.0701
Largest diff. peak and hole	1.226 and –1.360 e. $Å^{-3}$

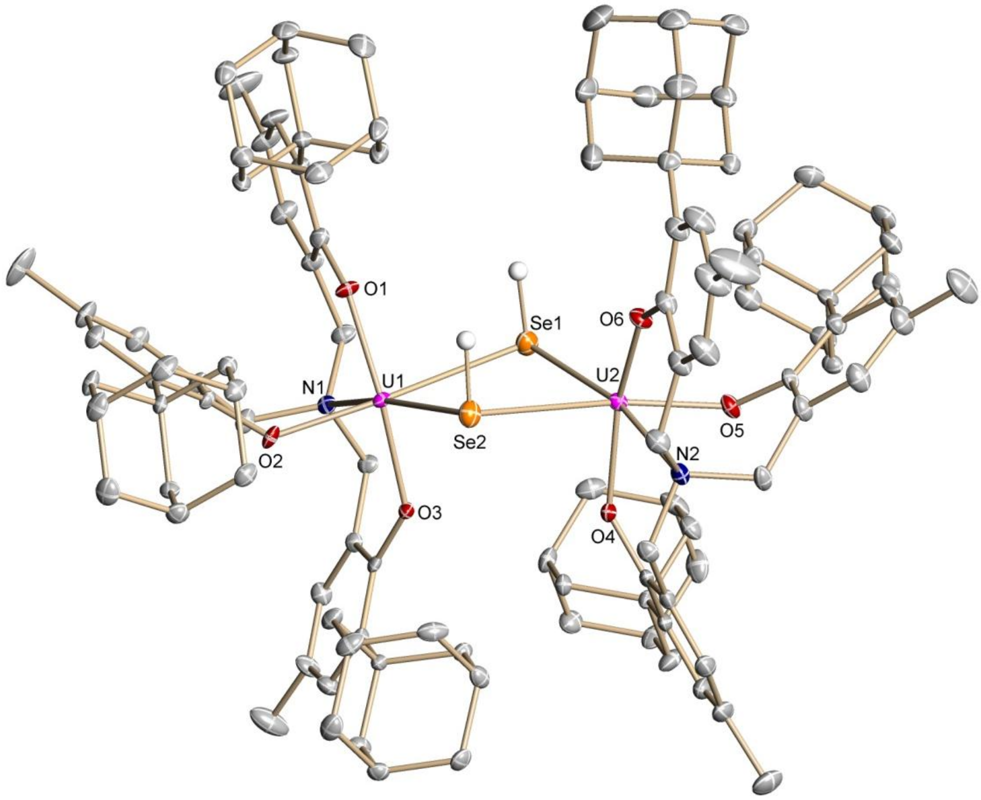

Figure S4. Molecular structure of **2-SeH** (top) and **2-TeH A** (bottom) with atom numbering scheme. Non-chalcogen bound H-atoms and co-crystallized solvent molecules are omitted for clarity. Thermal ellipsoids are at 50% probability.

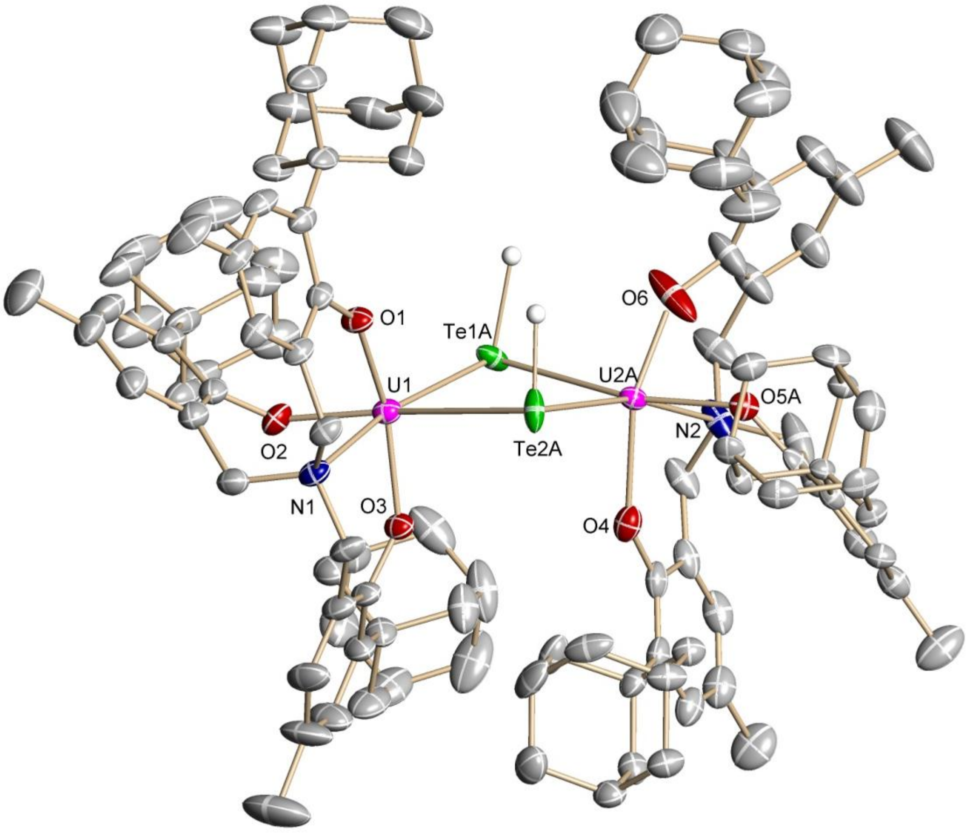

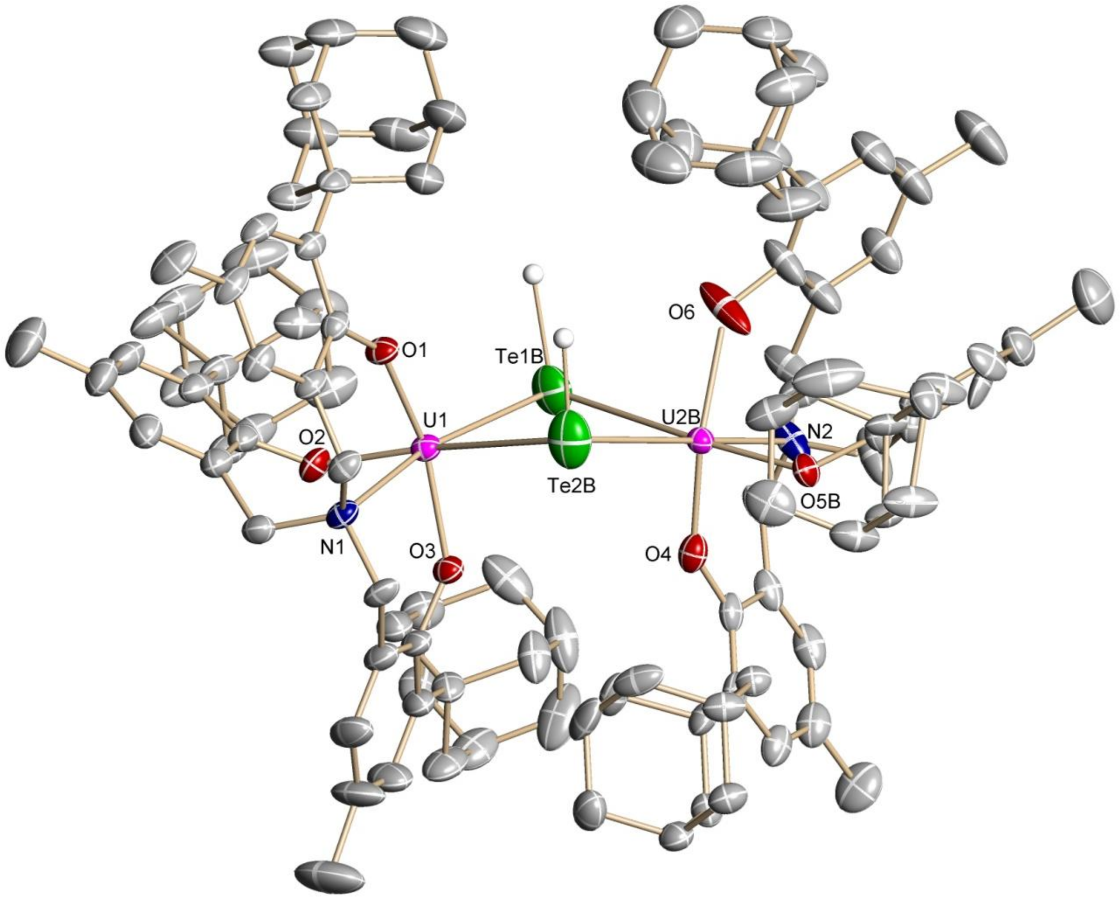

Figure S5. Molecular structure of **2-TeH B** with atom numbering scheme. Non-chalcogen bound H-atoms and co-crystallized solvent molecules are omitted for clarity. Thermal ellipsoids are at 50% probability.

Table 2. Structural Parameters for the two refined versions of **2-TeH**.

Structural Parameters	1-TeH A	1-TeH B
U–$O_{avg.}$	2.119, 2.136	2.119, 2.129
U–N	2.558(3), 2.567(3)	2.558(3), 2.529(3)
U_1–$E_{1,2}$	3.145(2), 3.296(2)	3.192(2), 3.285(2)
U_2–$E_{1,2}$	3.163(2), 3.119(2)	3.372(2), 3.178(2)
N–U–E	171.00(7), 87.43(8)<br>94.94(9), 166.68(10)	169.74(8), 86.58(8)<br>87.36(10), 173.64(10)

Crystal data and structure refinement for 2-TeH.

Identification code	sf06
Empirical formula	C150 H179.50 N2 O6.35 Te2 U2
Formula weight	2843.32 g/mol
Temperature	150(2) K
Wavelength	0.71073 Å
Crystal system, space group	Triclinic, P-1
Unit cell dimensions	a = 16.7730(6) Å α = 105.196(6)°<br>b = 16.9230(16) Å β = 102.968(7)°<br>c = 25.039(3) Å γ = 101.507(5)°
Volume	6426.8(9) $Å^3$
Z, Calculated density	2, 1.469 Mg/m^3
Absorption coefficient	3.019 mm^{-1}
F(000)	2865
Crystal size	0.40 x 0.19 x 0.14 mm^3
Theta range for data collection	2.82 to 27.10°
Limiting indices	–21<=h<=21, –21<=k<=21, –32<=l<=32
Reflections collected / unique	160728 / 28322 [R(int) = 0.0421]
Completeness to theta = 27.00	99.8 %
Absorption correction	Semi-empirical from equivalents
Max. and min. transmission	0.746 and 0.532
Refinement method	Full-matrix least-squares on F^2
Data / restraints / parameters	28322 / 419 / 1661
Goodness-of-fit on F^2	1.165
Final R indices [I>2sigma(I)]	R1 = 0.0360, wR2 = 0.0750
R indices (all data)	R1 = 0.0550, wR2 = 0.0839
Largest diff. peak and hole	1.269 and –0.799 e. $Å^{-3}$

^{1}H NMR Spectroscopy

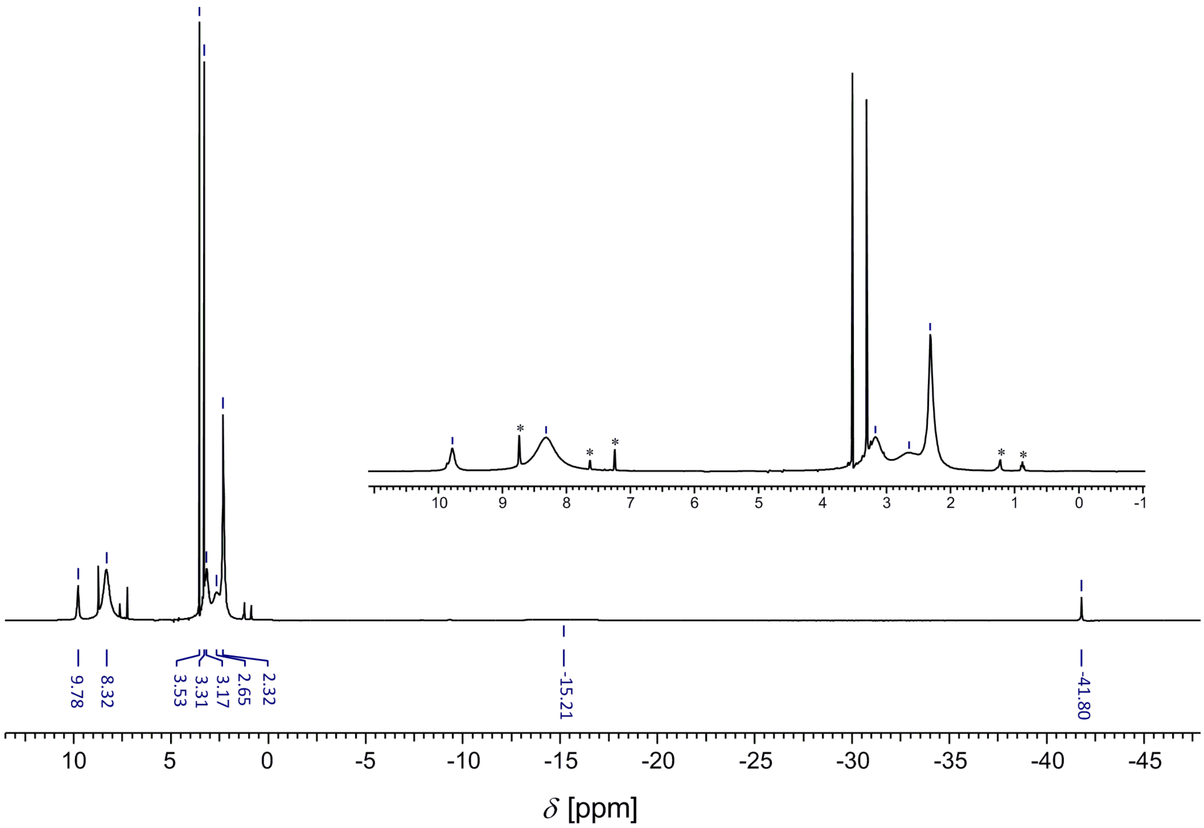

Figure S6. ^{1}H NMR spectrum of **1-SH** recorded in pyridine-d_5.

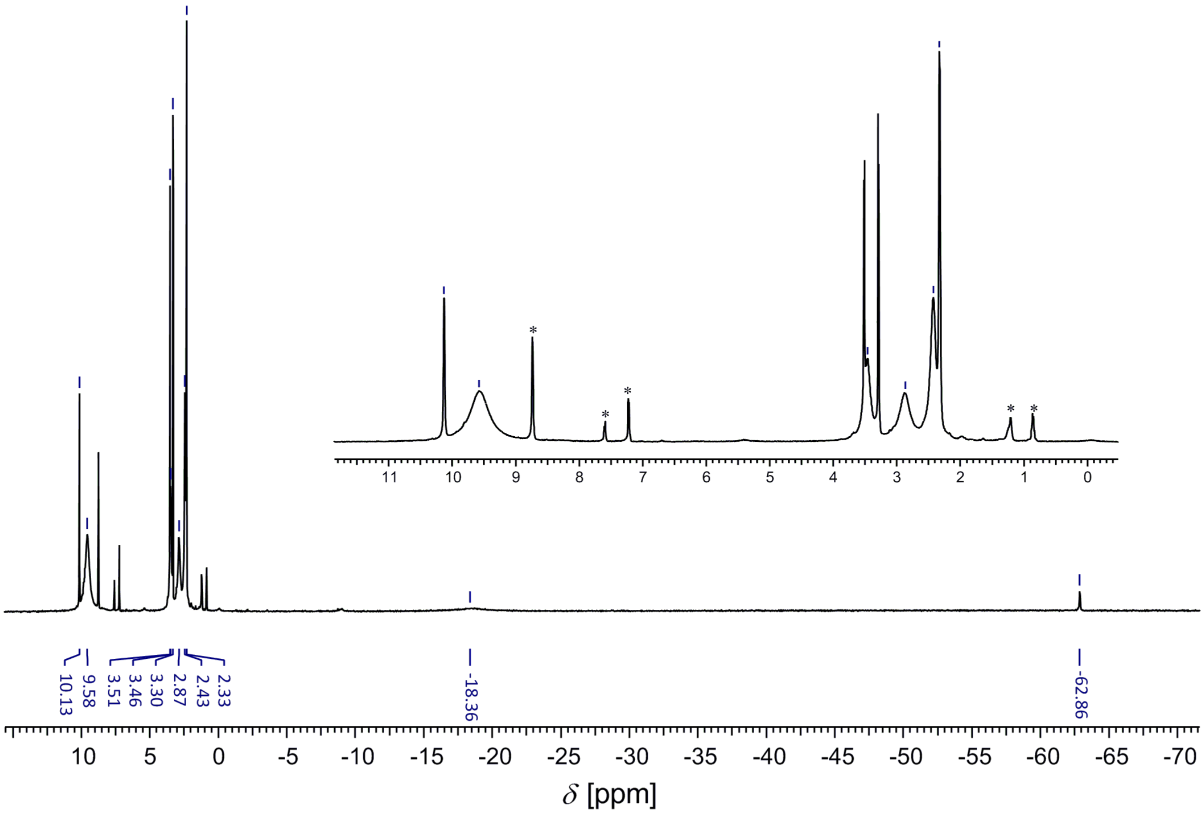

Figure S7. ^{1}H NMR spectrum of **1-SeH** recorded in pyridine-d_5.

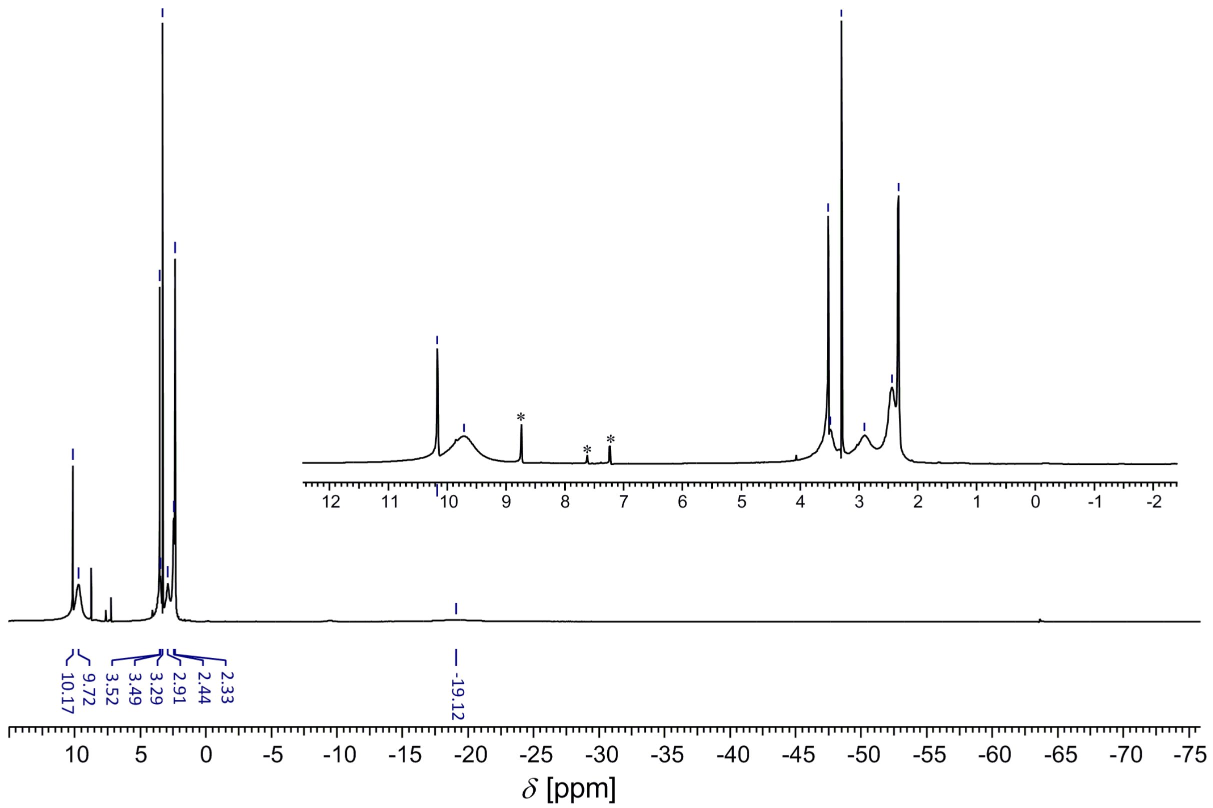

Figure S8. ^{1}H NMR spectrum of **1-SeD** recorded in pyridine-d_5.

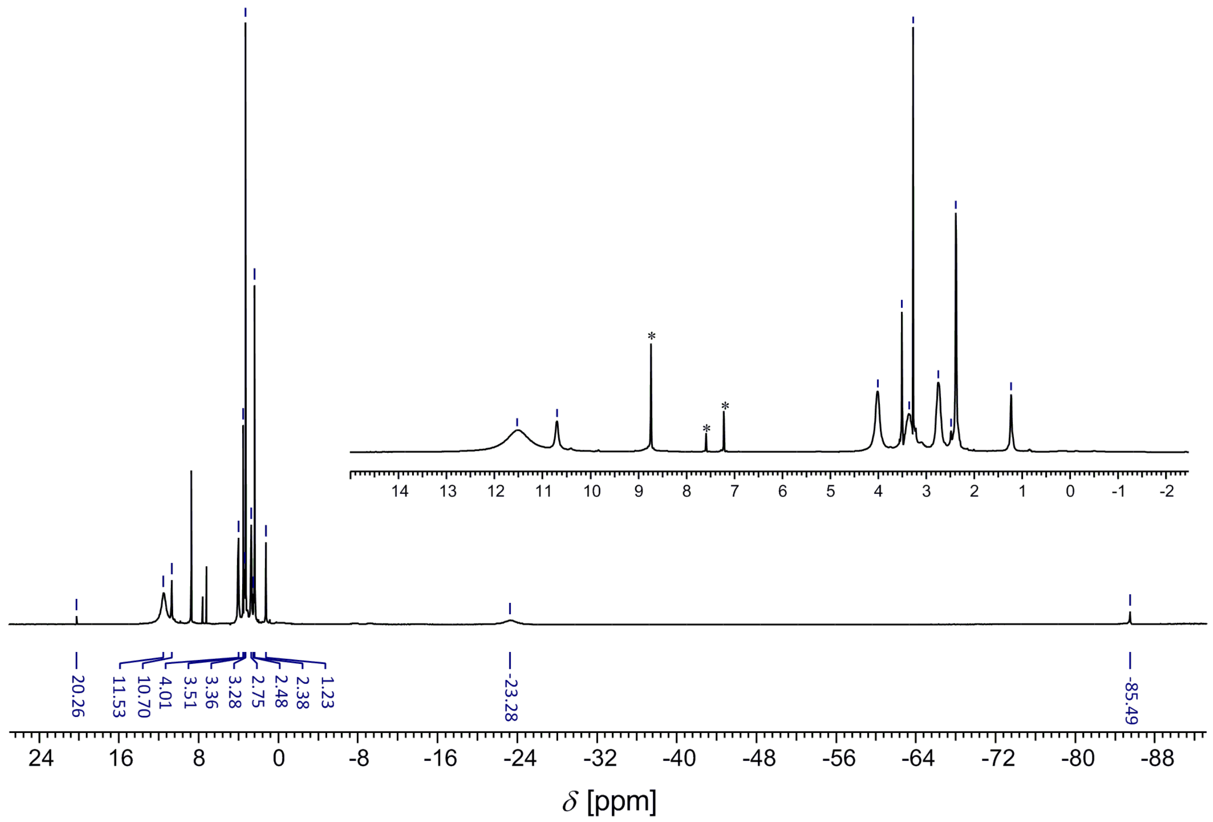

Figure S9. ^{1}H NMR spectrum of **1-TeH** recorded in pyridine-d_5.

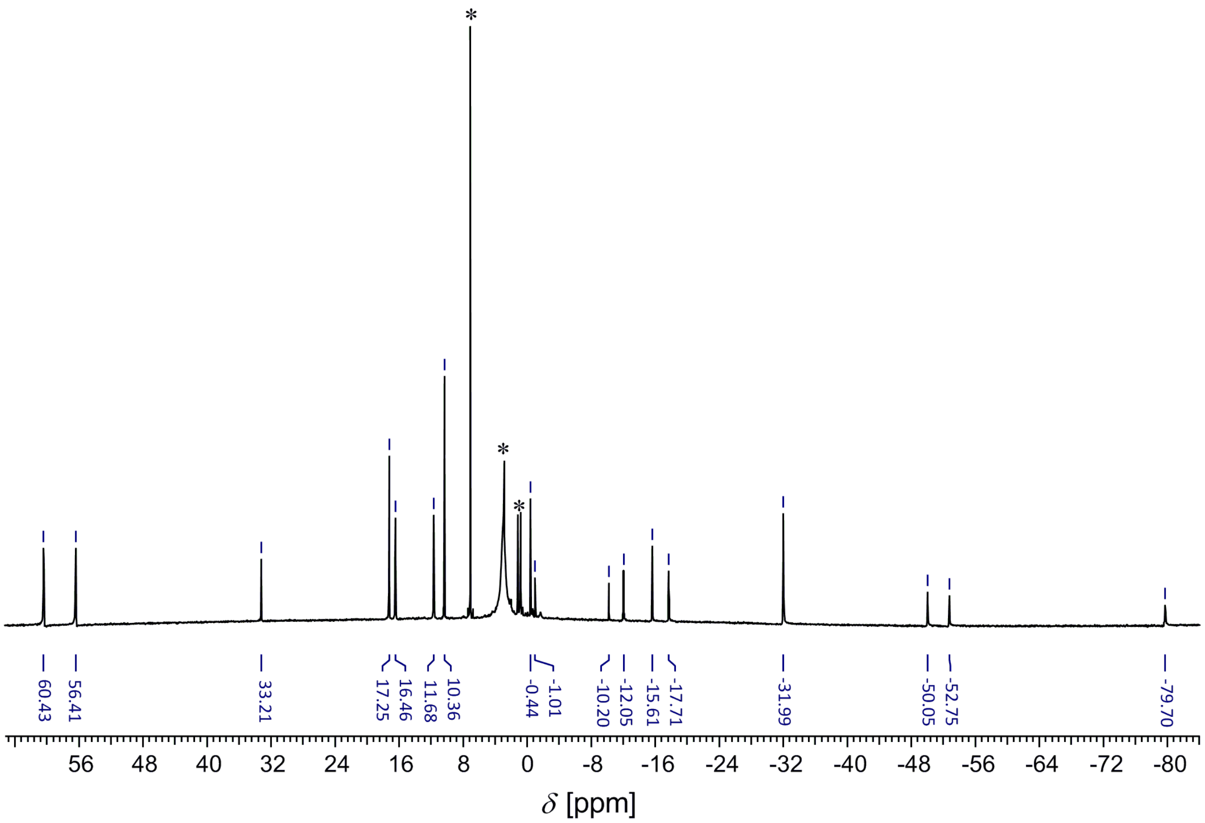

Figure S10. ^{1}H NMR spectrum of **2-SH** recorded in benzene-d_6.

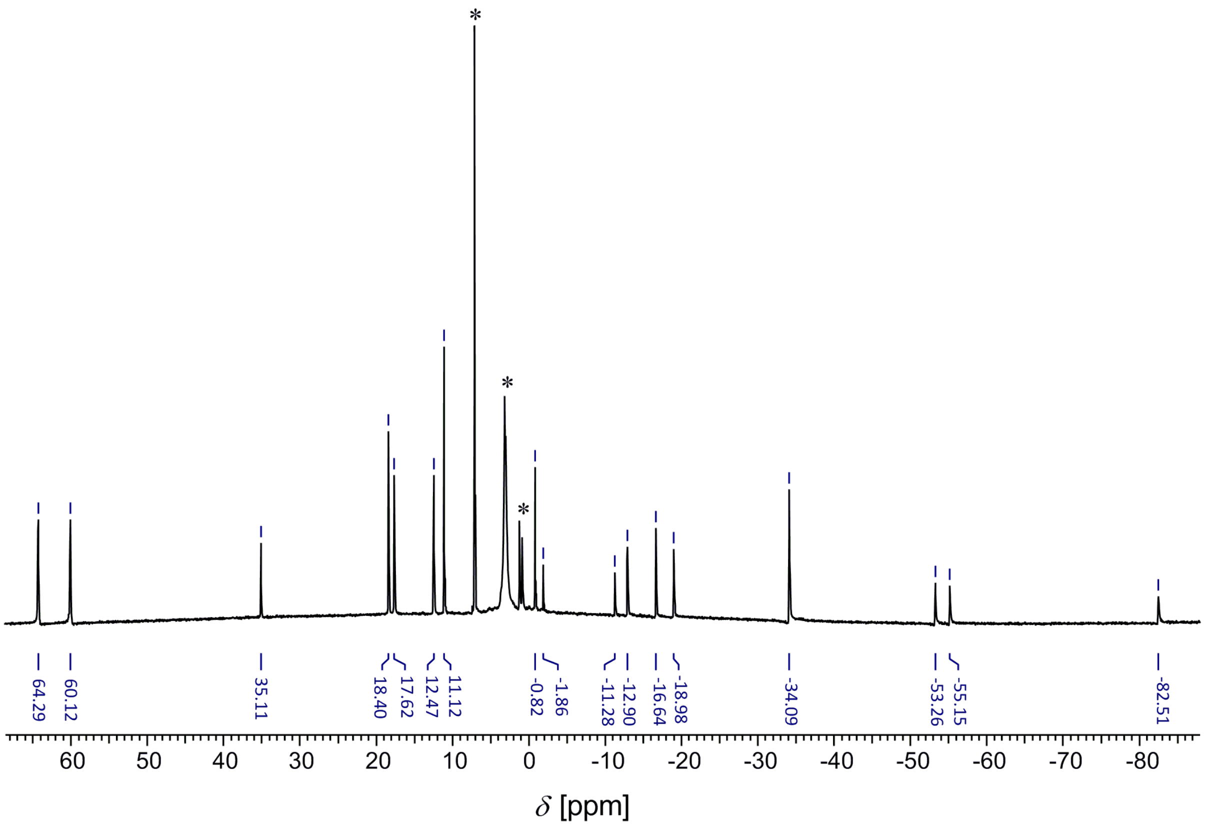

Figure S11. ^{1}H NMR spectrum of **2-SeH** recorded in benzene-d_6.

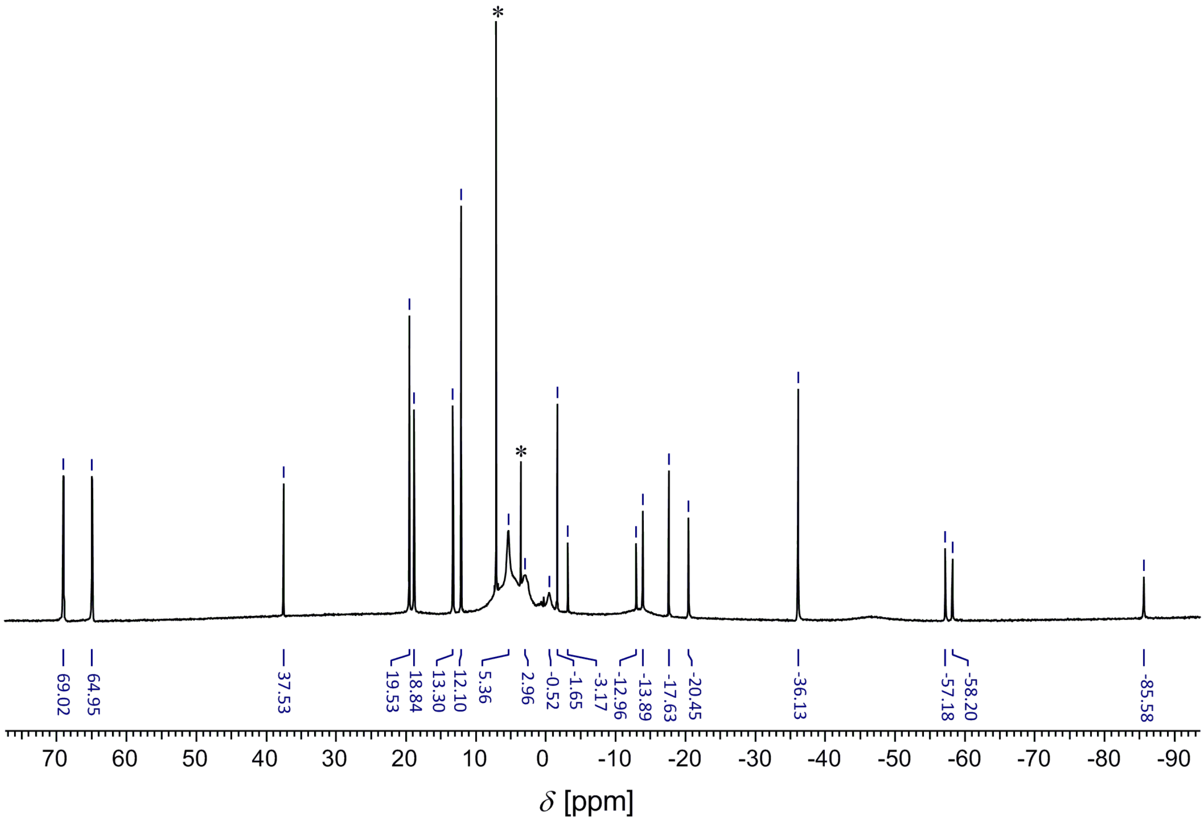

Figure S12. ^{1}H NMR spectrum of **2-TeH** recorded in benzene-d_6.

^{2}D NMR spectroscopy

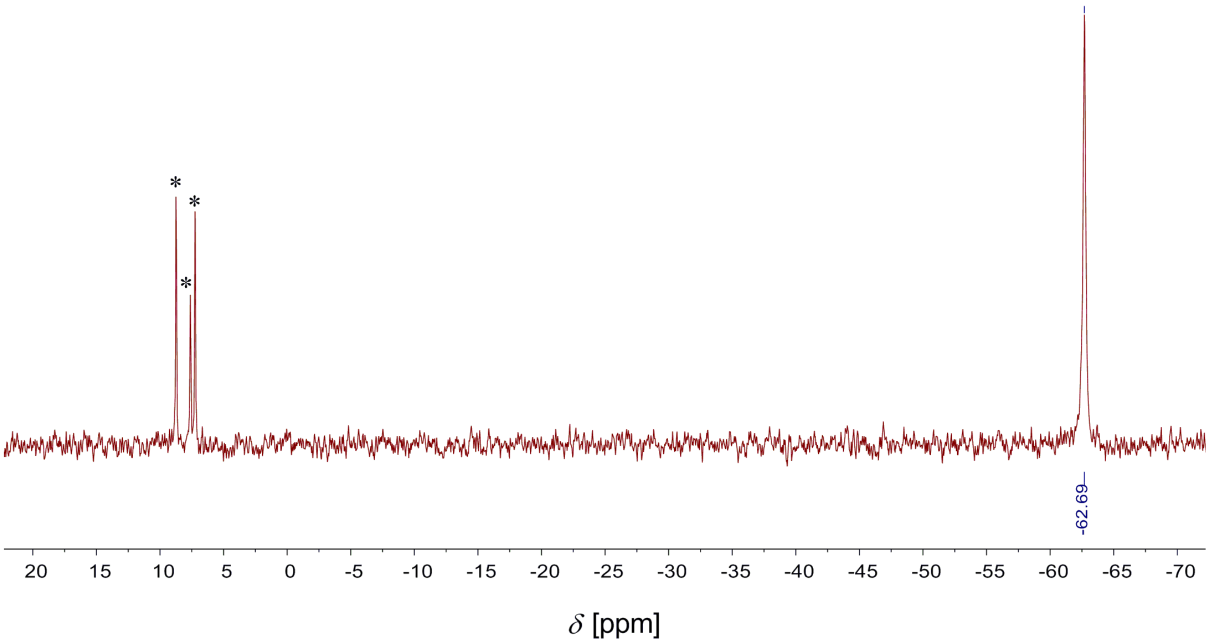

Figure S13. ^{2}H{^{1}H} NMR spectrum of **1-SeD** recorded in pyridine.

^{77}Se NMR Spectroscopy

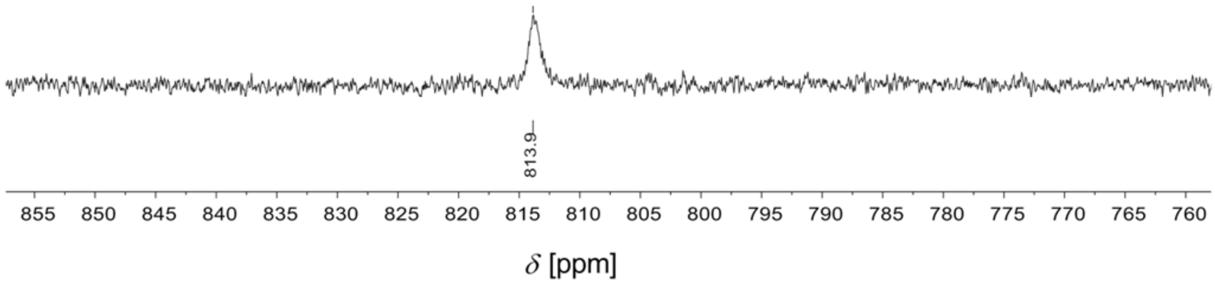

Figure S14. ^{77}Se NMR spectrum of **1-SeH** recorded in pyridine-d_5.

^{125}Te NMR Spectroscopy

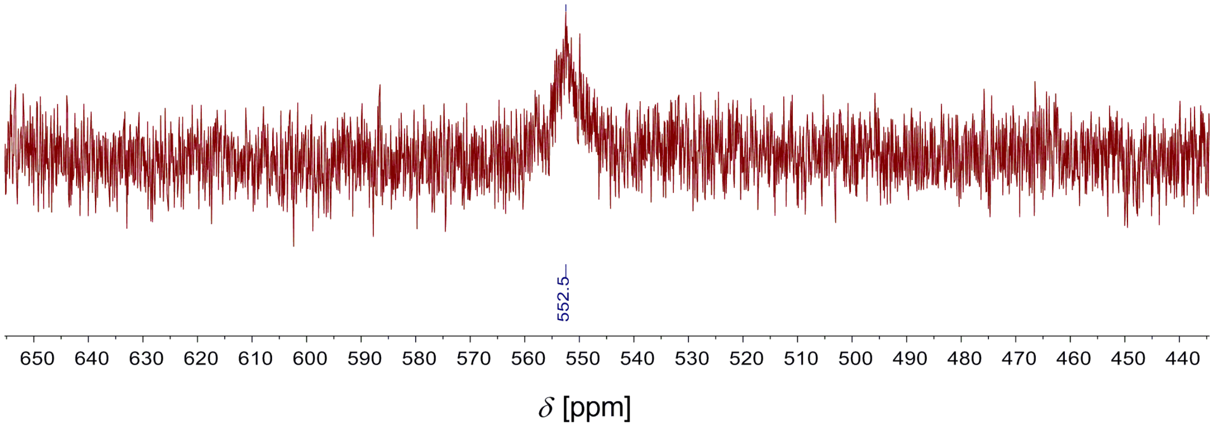

Figure S15. ^{125}Te NMR spectrum of **1-TeH** recorded in pyridine-d_5.

SQUID Magnetization Measurements

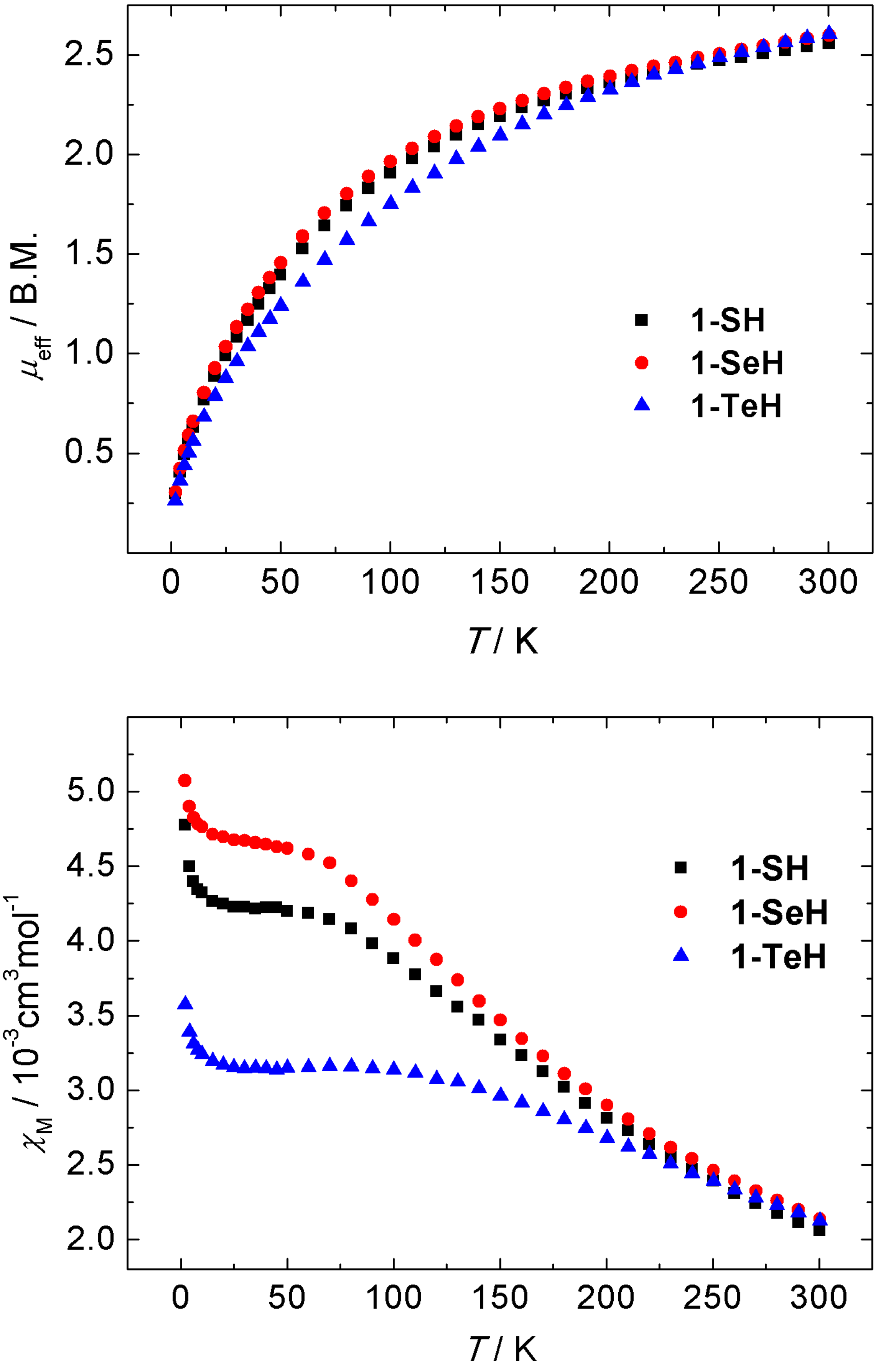

Figure S16. Temperature-dependent SQUID magnetization data of hydrochalcogenido complexes **1-EH** (E = S (black), Se (red), Te (blue)) as a plot of μ_{eff} *vs.* T (top) and χ_M *vs.* T (bottom). Data were corrected for underlying magnetism. Reproducibility was checked by at least two independently synthesized and measured samples for each compound.

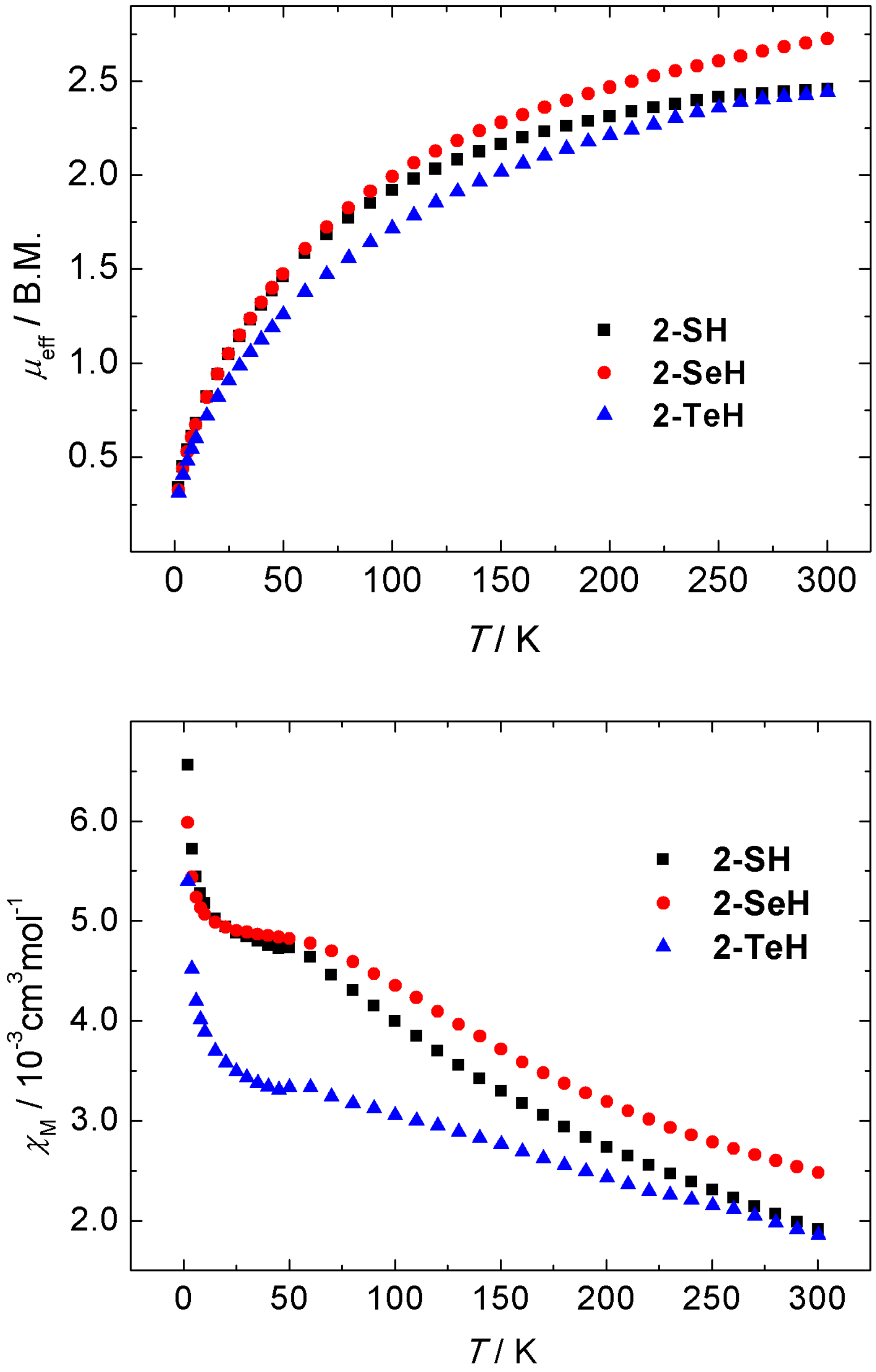

Figure S17. Temperature-dependent SQUID magnetization data of hydrochalcogenido complexes **2-EH** (E = S (black), Se (red), Te (blue)) as a plot of μ_{eff} *vs.* T (top) and χ_M *vs.* T (bottom). Data were corrected for underlying magnetism. Reproducibility was checked by at least two independently synthesized and measured samples for each compound.

IR Spectroscopy

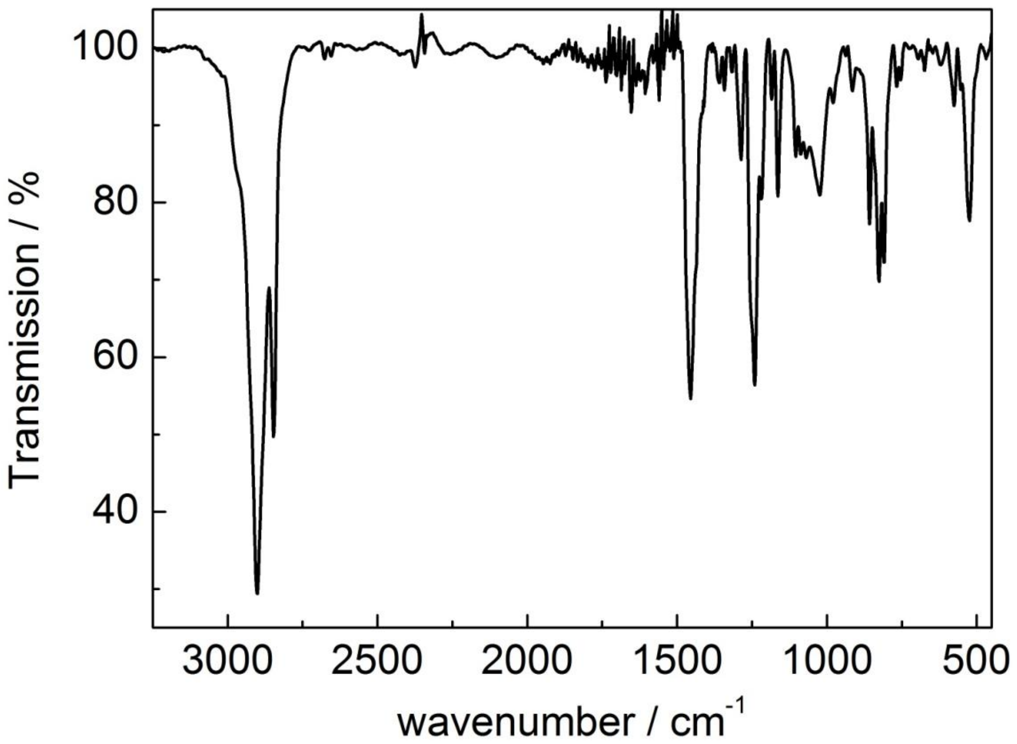

Figure S18. IR spectrum of **1-SH** recorded in KBr.

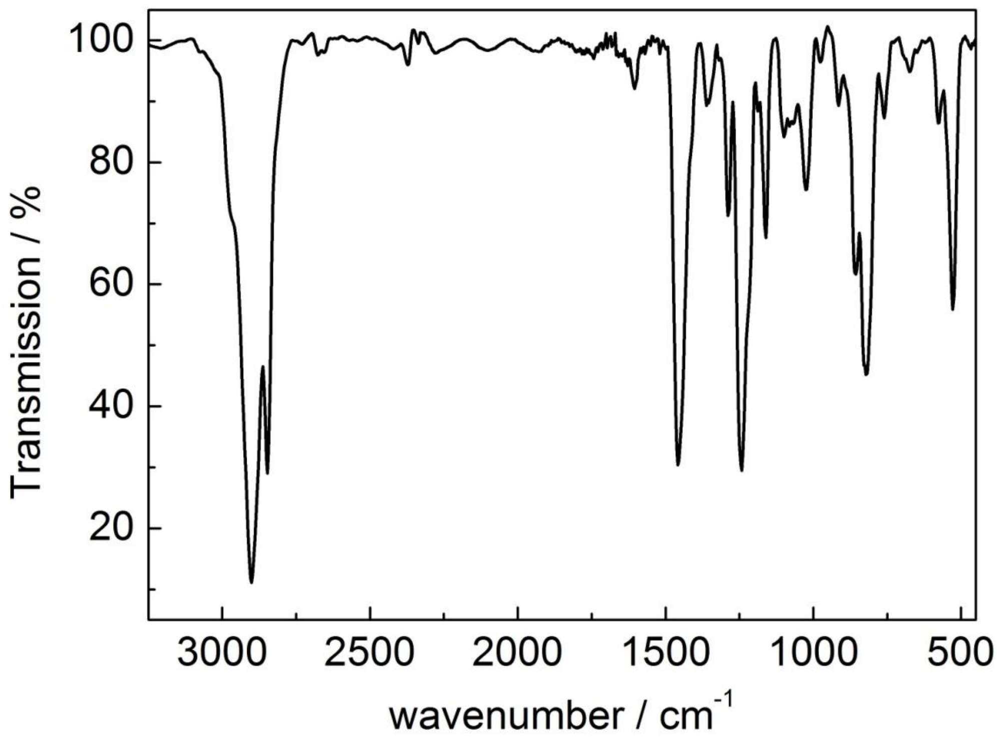

Figure S19. IR spectrum of **1-SeH** recorded in KBr.

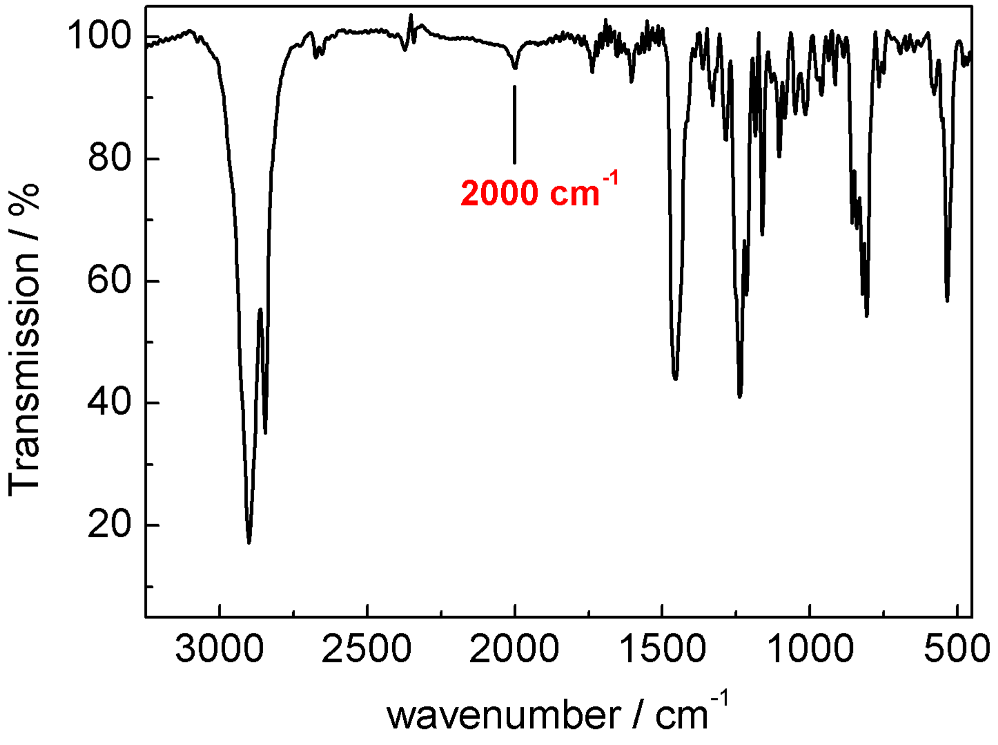

Figure S20. IR spectrum of **1-TeH** recorded in KBr.

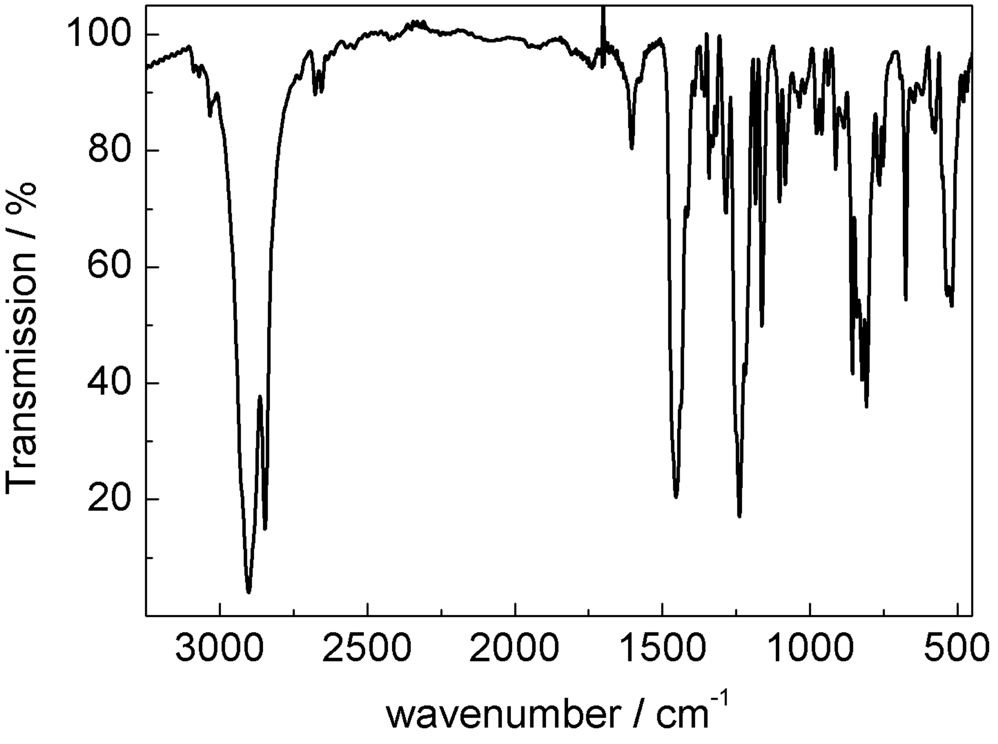

Figure S21. IR spectrum of **2-SH** recorded in KBr.

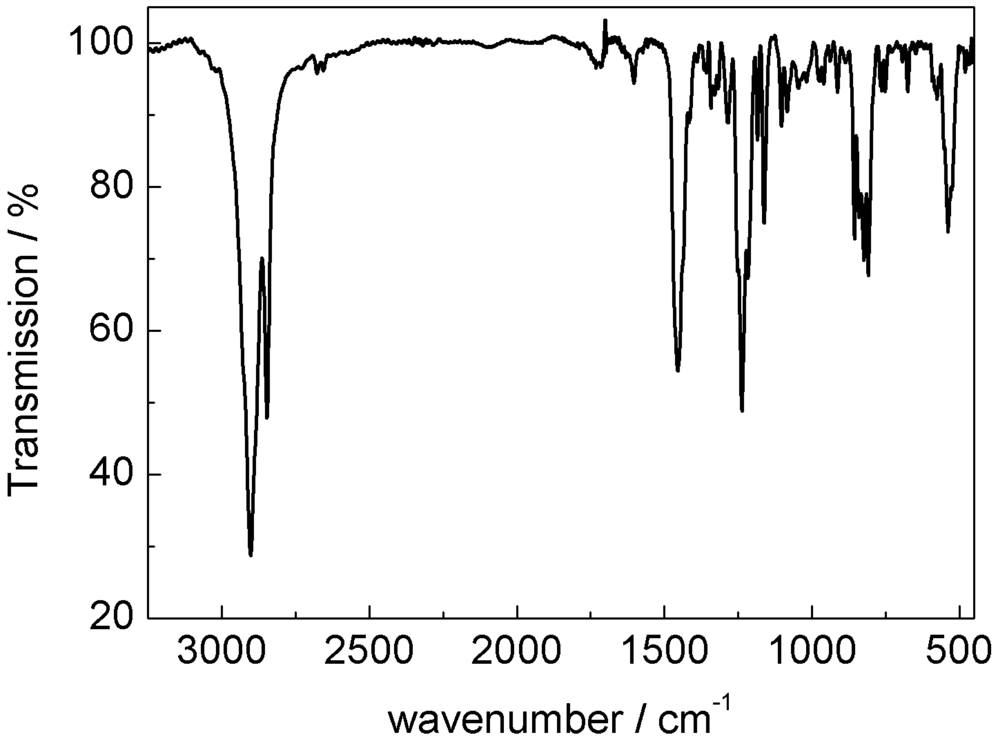

Figure S22. IR spectrum of **2-SeH** recorded in KBr.

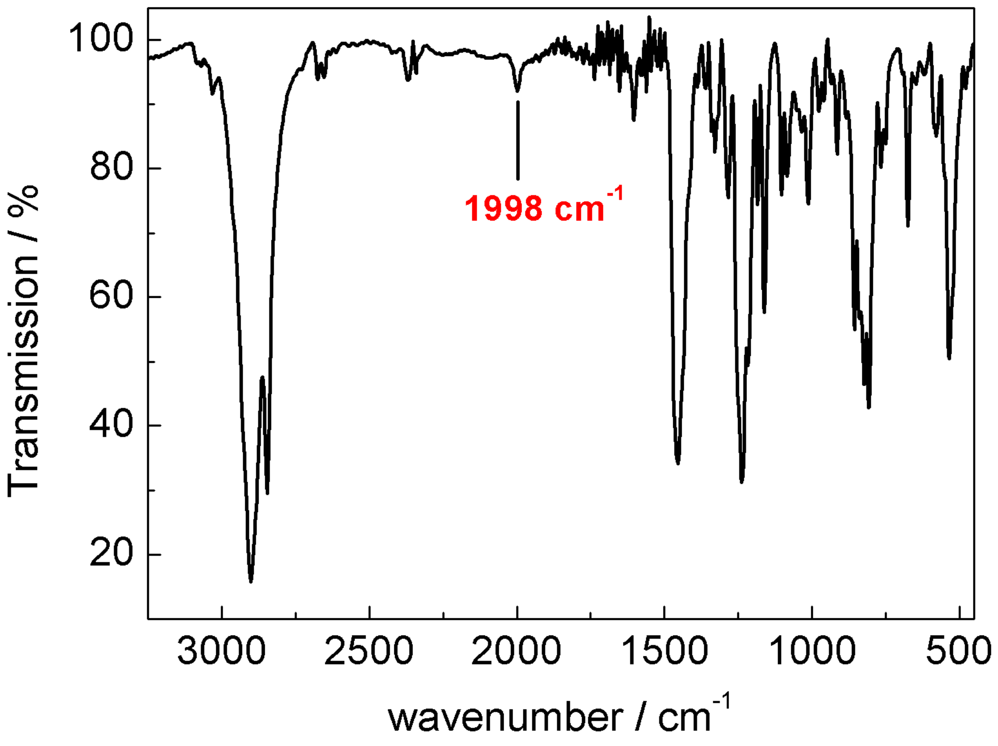

Figure S23. IR spectrum of **2-TeH** recorded in KBr.

UV/vis Spectroscopy

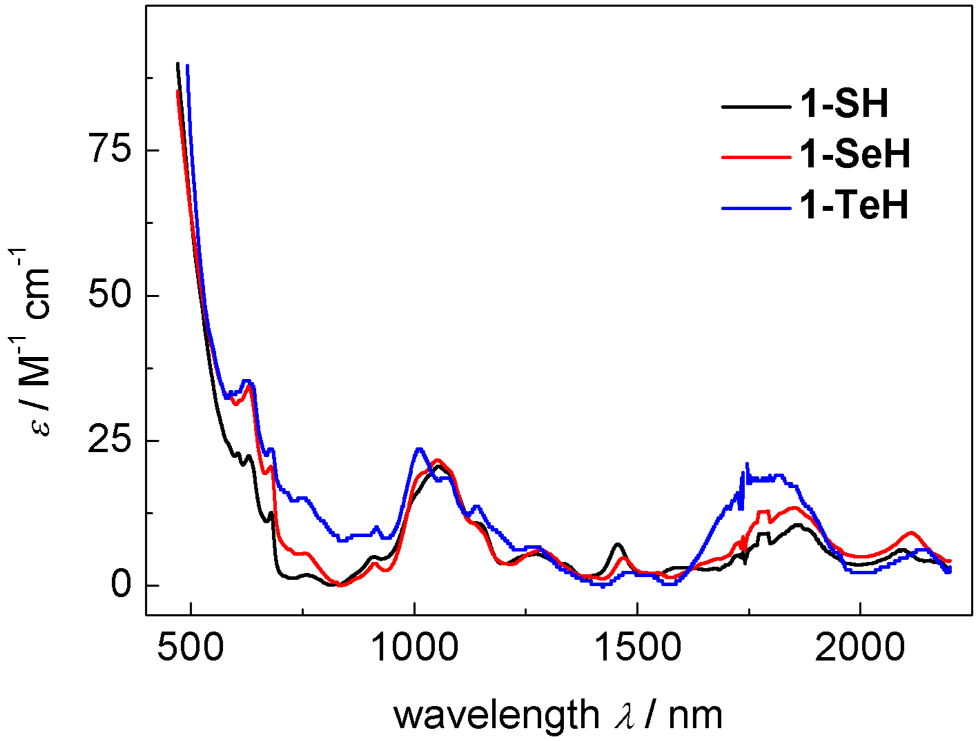

Figure S24. Vis/NIR spectra of hydrochalcogenido complexes **1-SH** (9.2 mM, black), **1-SeH** (9.7 mM, red), and **1-TeH** (4.1 mM, blue) recorded in DME at 25 °C.

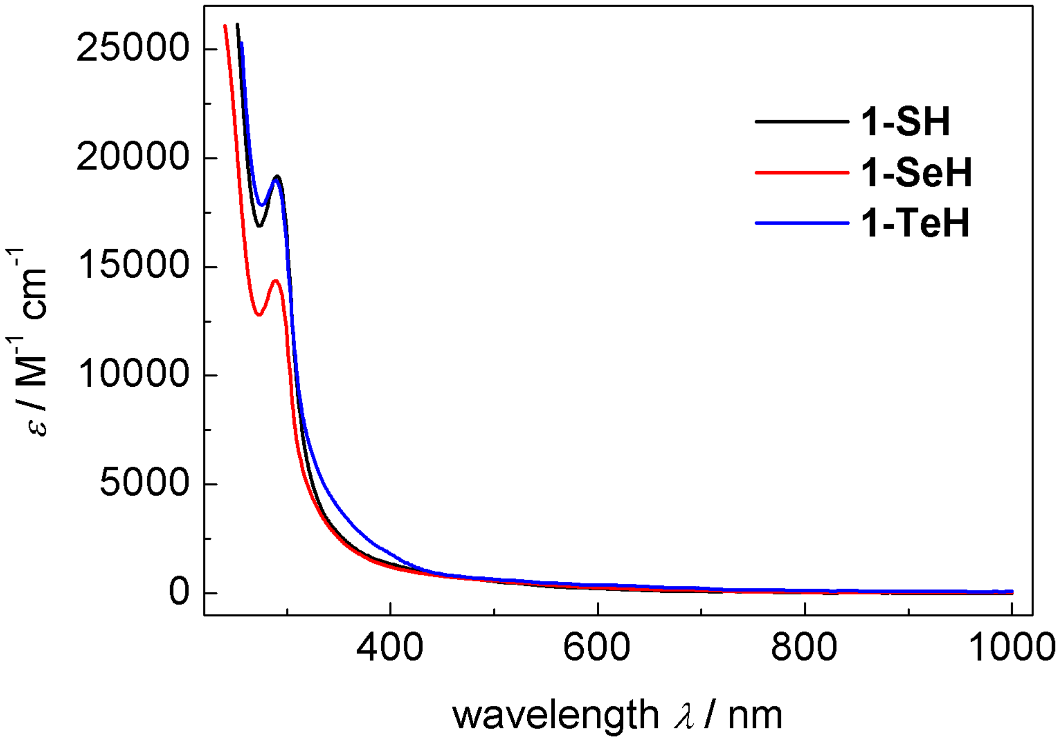

Figure S25. UV/vis spectra of hydrochalcogenido complexes **1-SH** (3.7×10^{-2} mM, black), **1-SeH** (9.7×10^{-2} mM, red), and **1-TeH** (4.1×10^{-2} mM, blue) recorded in DME at 25 °C.

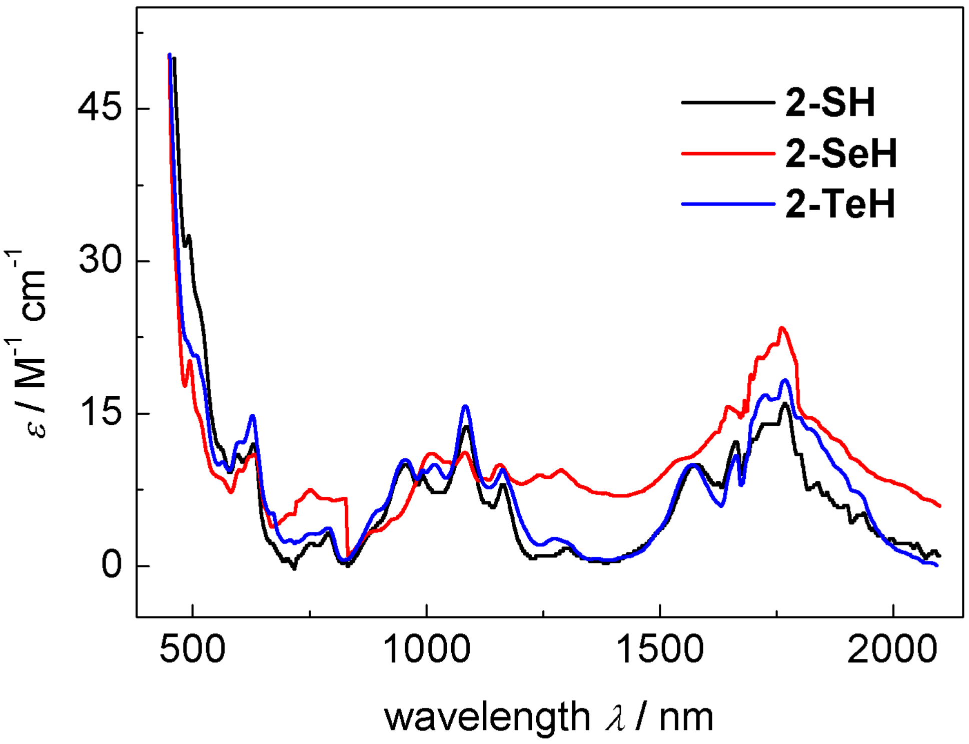

Figure S26. Vis/NIR spectra of hydrochalcogenido complexes **2-SH** (2.0 mM, black), **2-SeH** (6.6 mM, red), and **2-TeH** (6.5 mM, blue) recorded in benzene at 25 °C.

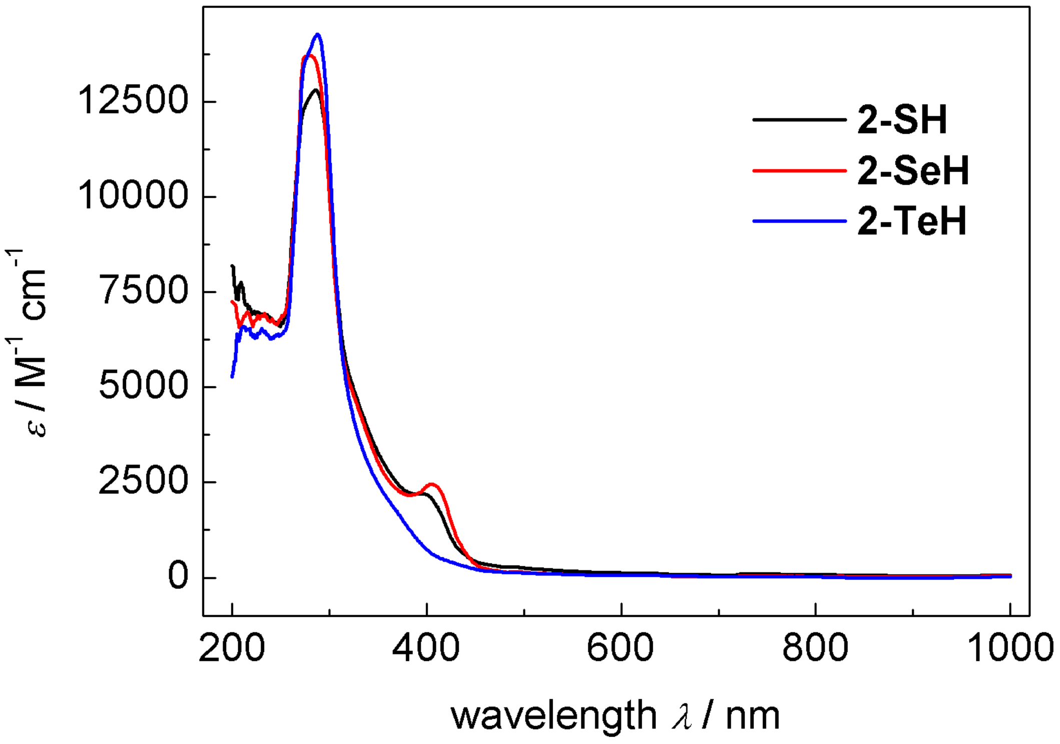

Figure S27. UV/vis spectra of hydrochalcogenido complexes **2-SH** (5.5×10^{-2} mM, black), **2-SeH** (6.6×10^{-2} mM, red), and **2-TeH** (6.5×10^{-2} mM, blue) recorded in benzene at 25 °C.

Cyclic Voltammetry

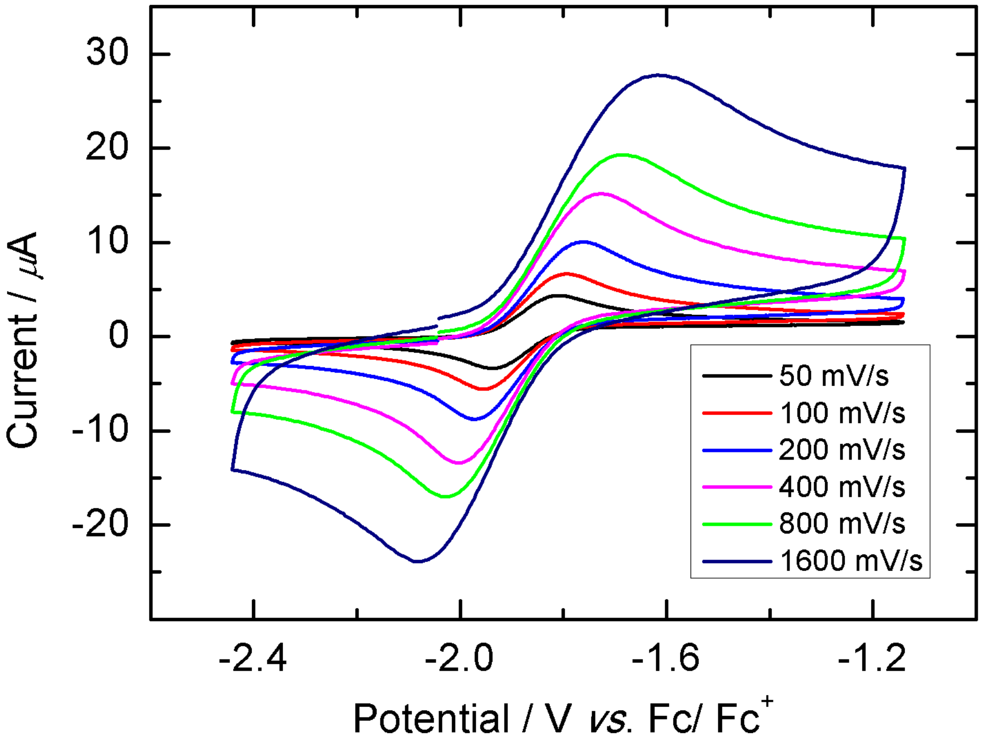

Figure S28 Cyclic voltammogram of uranium(III) starting material [((^{Ad}ArO)$_3$N)U(DME)] recorded at different scan rates in ~0.1 M [N(*n*-Bu)$_4$][BPh$_4$] in THF at room temperature.

References

1. G. A. Bain and J. F. Berry, *J. Chem. Educ.*, 2008, **85**, 532-536.
2. M. J. Monreal, R. K. Thomson, T. Cantat, N. E. Travia, B. L. Scott and J. L. Kiplinger, *Organometallics*, 2011, **30**, 2031-2038.
3. S. A. Cortes, M. A. Muñoz Hernández, H. Nakai, I. Castro-Rodriguez, K. Meyer, A. R. Fout, D. L. Miller, J. C. Huffman and D. J. Mindiola, *Inorg. Chem. Commun.*, 2005, **8**, 903-907.
4. O. P. Lam, S. C. Bart, H. Kameo, F. W. Heinemann and K. Meyer, *Chem. Commun.*, 2010, **46**, 3137-3139.
5. M. Baudler, G. Brauer, F. Fehér, F. Huber, R. Klement, W. Kwasnik, P. W. Schenk, M. Schmeisser and R. Steudel, *Handbuch der präparativen anorganischen Chemie*, 3rd ed.; G. Brauer, Ed.; Ferdinand Enke Verlag, Stuttgart, Germany, 1975, Vol.1.
6. SADABS, *Bruker AXS Inc.*, 2008, Madison, U.S.A.
7. G. M. Sheldrick, *Acta Cryst.*, 2008, *A64*, 112 - 122.
